# Synthesis of Four Pentacyclic Triterpene–Sialylglycopeptide Conjugates and Their Affinity Assays with Hemagglutinin

**DOI:** 10.3390/molecules26040895

**Published:** 2021-02-08

**Authors:** Mei Luo, Ximin Wu, Yiming Li, Fujiang Guo

**Affiliations:** School of Pharmacy, Shanghai University of Traditional Chinese Medicine, Shanghai 201203, China; aluofor@126.com (M.L.); wxm@shutcm.edu.cn (X.W.)

**Keywords:** influenza virus, pentacyclic triterpenes, sialylglycopeptide, hemagglutinin

## Abstract

Influenza outbreaks pose a serious threat to human health. Hemagglutinin (HA) is an important target for influenza virus entry inhibitors. In this study, we synthesized four pentacyclic triterpene conjugates with a sialylglycopeptide scaffold through the Cu(I)-catalyzed alkyne-azide cycloaddition reaction (CuAAC) and prepared affinity assays of these conjugates with two HAs, namely H1N1 (A/WSN/1933) and H5N1 (A/Hong Kong/483/97), respectively. With a dissociation constant (*K*_D_) of 6.89 μM, SCT-Asn-betulinic acid exhibited the strongest affinity with the H1N1 protein. Furthermore, with a *K*_D_ value of 9.10 μM, SCT-Asn-oleanolic acid exhibited the strongest affinity with the H5N1 protein. The conjugates considerably enhanced antiviral activity, which indicates that pentacyclic triterpenes can be used as a ligand to improve the anti-influenza ability of the sialylglycopeptide molecule by acting on the HA protein.

## 1. Introduction

Influenza is a highly contagious acute upper respiratory disease caused by influenza viruses. Seasonal influenza causes 29–65 million deaths annually [1], and large outbreaks can be even more severe [2]. Current prevention and treatment methods mainly include vaccines and drugs. However, antiviral drugs are typically used for treatment because vaccines have a long development time, high cost, and reduced effectiveness resulting from antigen drift [3]. Currently, depending on the glycoproteins and enzymes involved when the virus infects the host cell [4], the main targets include hemagglutinin (HA), matrix proteins (M1), proton ion channel proteins (M2), RNA-dependent RNA polymerase (RdRp), nucleoprotein (NP), nonstructural protein 1 (NS1), nuclear export protein (NS2), neuraminidase (NA), and polymerase proteins (PB1, PB2 and PA) [5]. Two types of drugs have been developed to hinder influenza virus infection. Oseltamivir, zanamivir, and peramivir act on NA, and amantadine and rimantadine act on the M2 ion channel protein. However, new antiviral drugs should be continuously developed because of drug side effects and viral resistance [6,7].

HA, a trimer, is composed of HA1-containing sialic acid (SA) binding sites and HA2 anchored on the surface of the virus [8]. HA1 mediates attachment to SA receptors on the target cell, and HA2 undergoes extensive conformational changes, which contribute to membrane fusion and the release of viral genetic materials into host cells [9]. According to the results of a study on antigen profiles, with the exception of the SA receptor-binding domain, mutations in HA1 are tolerable without affecting the overall function of HA [10]. Thus, HA is a potential target for antiviral drugs.

Sialylglycopeptide (SGP), with two SA fragments at the molecule end, is a form in which SA exists as a glycopeptide [11] and can be prepared in large quantities from egg yolk [12]. Studies have revealed that in influenza A virus infection, HA first binds to the NeuAcα(2–6) Gal residues of the sugar chain on the host cell surface [13]. SGP with this residue has studied widely studied; Bovin et al. reported the effective inhibitory effect of the SGP polymer with polyacrylamide on influenza virus infection [14]. Considering the safety of polyacrylamide for host cells, chitosan—which is a safe biocompatible material—is used to synthesize chitosan-SGP complexes [15]. SGP is also used to form conjugates with human serum albumin [16], glycopolymers [17], and erythropoietin [18]. SGP has a representative N-sugar chain structure for protein glycosylation. This chain is a vital intermediate for the synthesis of glycopeptides and glycoproteins with a single glycoform structure [19,20,21,22,23,24], and it can avoid complex synthesis processes [25,26]. Grafting SA to other biologically active molecules, such as triterpenes, can also increase antiviral activity [27,28]. Xiao et al. investigated the synthesis of conjugates of pentacyclic triterpenes linked to SA at various sites and its effect on antiviral activity [29]. Pentacyclic triterpenes, which can be widely found in Chinese herbal medicine [30], are a class of secondary metabolites [31] with anti-influenza capability [32,33,34]. In addition to combining with SA, pentacyclic triterpenes can be conjugated with small molecules, such as oligosaccharides [35,36] or L-ascorbic acid [37]—and other macromolecules, such as cyclodextrin [38], methylated cyclodextrin [39], and human serum albumin [40]—to form multivalent copolymers, which considerably improve antiviral activity. These studies are meaningful supplements to heterocyclic compounds related to natural product synthesis, structural analysis, and biological activity research. Heterocyclic compounds not only have biological activity [41,42], but also make outstanding contributions in the field of catalytic reactions [43] and as natural product extractants [44,45]. In our previous work, SGP was hydrolyzed to obtain the glycan portion (SCT-Asn). Surface plasmon resonance (SPR) revealed that SCT-Asn exhibits a moderate affinity to HA, whereas SGP exhibits no affinity to it. Therefore, we modified the SCT-Asn structure and used pentacyclic triterpenes as ligands to synthesize conjugates. We then preliminarily assayed the affinity of conjugates to the HA protein through SPR.

## 2. Results and Discussion

### 2.1. Chemistry

**Figure 1:**
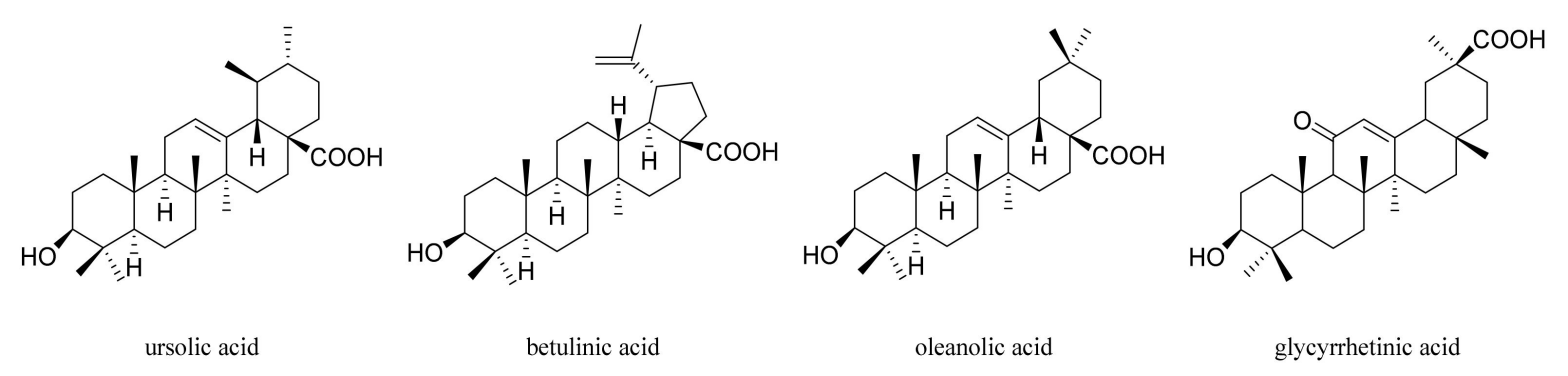
Chemical structures of four parent pentacyclic triterpenes.

Four pentacyclic triterpenes, namely ursolic acid (UA, ursane type), betulinic acid (BA, lupane type), oleanolic acid (OA, oleanane type), and glycyrrhetinic acid (GA, oleanane type) were selected as ligands (Figure 1). We assembled four oligosaccharide-carrying pentacyclic triterpene conjugates and evaluated their influenza activities by testing their affinity with HA.

**Scheme 1:**
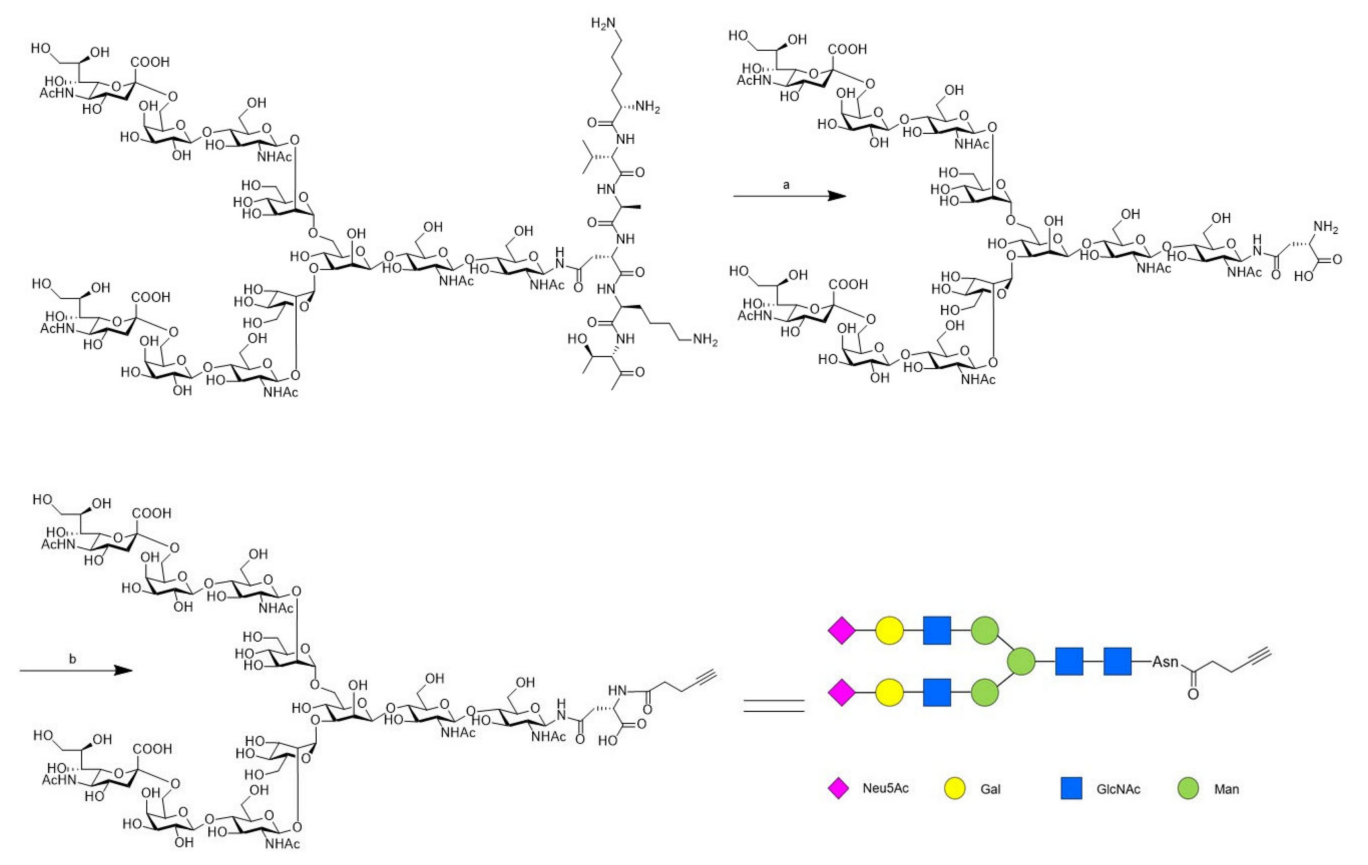
Synthesis of SCT-Asn-alkyne: (**a**) pronase, 0.5% SDS, 10 mM CaCl_2_, 0.1 M Tris, pH 7.5, 37 °C, 7 d; (**b**) pent-4-ynoic acid succinimidyl ester, H_2_O/DMF (1/1 *v/v*), RT, 12 h.

Here, 10 g of SGP was obtained from 20 kg of egg yolk powder using a method described in the literature [12]. First, alkyne-labeled N-glycan was prepared (Scheme 1). SGP was then thoroughly digested with pronase to remove its peptide portion, leaving asparagine (Asn)-linked N-glycans (SCT-Asn). The process was monitored through high performance liquid chromatography (HPLC) coupled with an ultraviolet detector. The retention time (*t*_R_) of SCT-Asn was 13.8 min, and it was well separated from the SGP peak (*t*_R_ = 21.7 min) under the same chromatographic conditions. After the digestion was complete, the residue was subjected to size-exclusion chromatography (Sephadex G-50) and lyophilized to obtain SCT-Asn (yield = 73.4%). The identity of SCT-Asn was confirmed through electrospray ionization mass spectrometry (ESI-MS) analysis.

To prepare SCT-Asn-alkyne, the obtained SCT-Asn was dissolved in a mixed solvent of water and dimethylformamide (DMF; 1/1 *v/v*). Next, the N-(4-pentynoyloxy)-succinimide synthesized in the previous step [46] was added, followed by triethylamine. The mixture was incubated at room temperature for 12 h. The residue was subjected to Sephadex G-25 for purification and lyophilized to obtain SCT-Asn-alkyne as a white solid (yield = 56.7%). The identity of SCT-Asn-alkyne was confirmed through ESI-MS analysis.

**Scheme 2:**
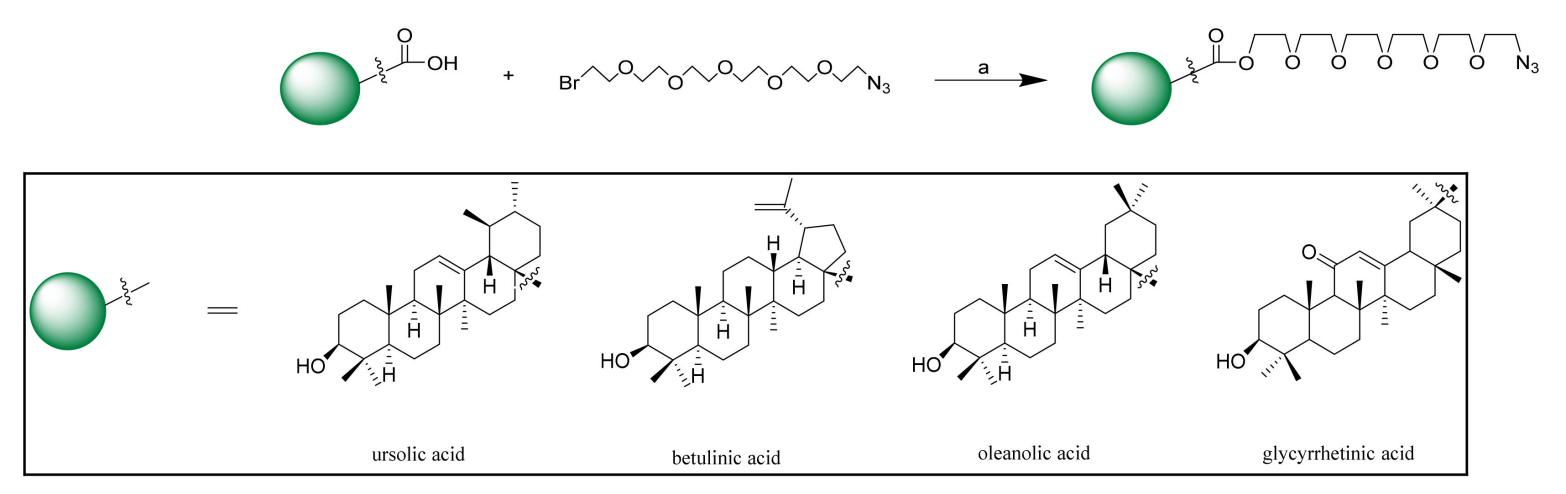
Synthesis of pentacyclic triterpene-N_3_: (**a**) K_2_CO_3_, DMF, RT, 18 h.

Second, azido-functionalized derivatives of pentacyclic triterpenes (ursolic acid-N_3_, betulinic acid-N_3_, oleanolic acid-N_3_, and glycyrrhetinic acid-N_3_) were synthesized through the esterification of C-28 or C-30 carboxylic acid with the azide group of Bromo-PEG5-azide in yields ranging from 67.6% to 76.6% (Scheme 2; the structures of pentacyclic triterpene-N_3_ are available in Supplementary File, Figure S1). The four compounds were confirmed through thin-layer chromatography (TLC), ESI-MS, and nuclear magnetic resonance (NMR).

**Scheme 3:**
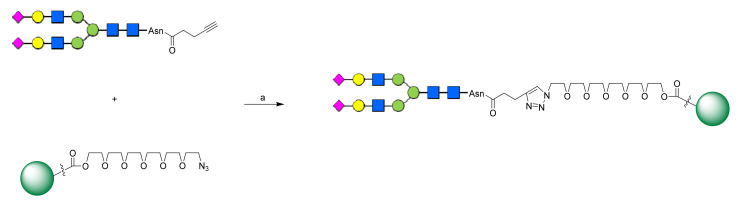
Synthesis of SCT-Asn-pentacyclic triterpene: (**a**) TBTA, sodium ascorbate, CuSO_4_, THF/H_2_O (3/2 *v/v*), RT, 2 h.

Finally, the target SCT-Asn-pentacyclic triterpene conjugates were synthesized, as displayed in Scheme 3. The conjugation of SCT-Asn-alkyne and pentacyclic triterpene-azide was achieved through the Cu(I)-catalyzed alkyne-azide cycloaddition reaction (CuAAC) [47]. A solution of the alkyne and azide in THF/H_2_O (3/2 *v/v*) was stirred with CuSO_4_, sodium ascorbate, and a copper ligand, tris(benzyltriazolylmethyl)amine (TBTA) [48], at room temperature. The residue was purified through column chromatography to obtain SCT-Asn-UA, SCT-Asn-BA, SCT-Asn-OA, and SCT-Asn-GA in yields ranging from 14.9% to 29.5% (the structures of SCT-Asn-pentacyclic triterpene are available in Supplementary File, Figure S2).

### 2.2. SPR Assay

SPR assay showed a certain effectiveness in determining the in vitro anti-influenza virus activity of biomolecules. Many studies have used SPR assay and other tests, such as the time-of-addition experiment, HA inhibition assay, molecular docking analysis, etc., to confirm the target and mechanism [27,35].

Biomolecules with SA residues exhibit certain anti-influenza virus activity. These biomolecules target HA. Therefore, when we investigated the anti-influenza virus activity of the compounds, we first tested its affinity with HA and determined its anti-influenza virus potential. The results could provide guidance for subsequent pharmacological experiments. In this study, we performed SPR assay of four synthesized conjugates with two types of HA, namely H1N1 (A/WSN/1933) and H5N1(A/Hong Kong/483/97), obtained from commercial sources.

The affinity of conjugates for HA was measured though SPR and expressed using the thermodynamic dissociation constant (*K*_D_). HA was immobilized on the surface of a CM5 chip by using an amine coupling approach. Conjugates at concentrations of 1.562–100 µM were injected, and the responses were measured. A Langmuir 1:1 binding model was fit using Biacore evaluation software (GE Healthcare) to determine the binding affinity.

Table 1 shows the results of SPR assay (sensorgrams of SPR assay; see Figures S3 and S4 in Supplementary File). The results revealed that the parent SGP exhibited no affinity with HA, whereas SCT-Asn exhibited enhanced affinity with *K*_D_ values of 29.04 and 75.46 μM for H1N1 and H5N1, respectively. The polypeptide portion of SGP weakens the interaction between SA residues and HA.

The results of binding to H1N1 proteins revealed that among the four pentacyclic triterpenes, OA exhibited the best affinity, with a *K*_D_ value of 31.14 μM, whereas BA exhibited no affinity. The affinity of UA and GA was marginally inferior, with *K*_D_ values of 136.70 and 584.00 µM, respectively. For H5N1, OA again exhibited the best affinity, with a *K*_D_ value of 47.78 µM, but the *K*_D_ value of GA, UA and BA exhibited no significant affinity.

**Figure 2:**
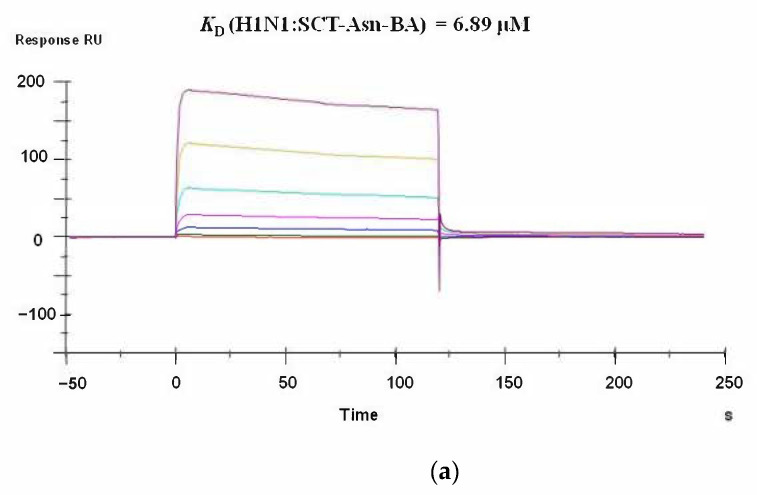

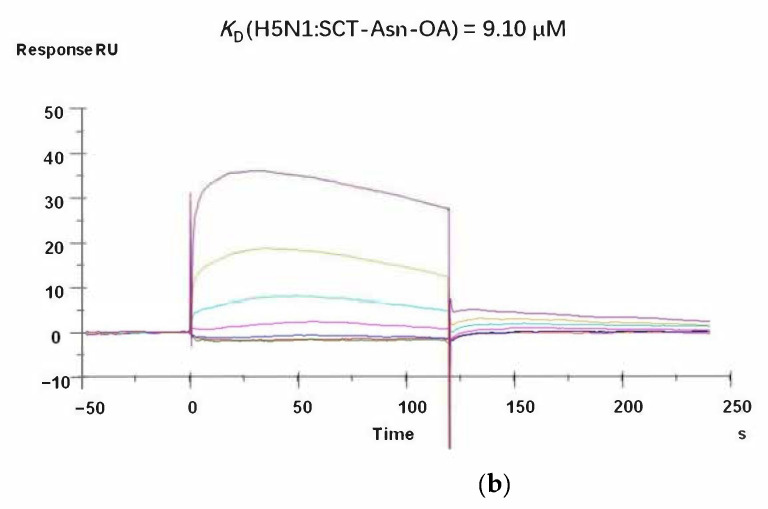
Surface plasmon resonance (SPR) assay characterization between conjugates and hemagglutinin proteins. (**a**) SCT-Asn-BA with H1N1(A/WSN/1933) protein; (**b**) SCT-Asn-OA with H5N1(A/Hong Kong/483/97) protein. Their *K*_D_ values are labeled in the corresponding curves.

When the pentacyclic triterpene was linked to SCT-Asn, three conjugates (all except for SCT-Asn-UA) exhibited enhanced affinity with H1N1. Among them, SCT-Asn–BA exhibited the best affinity, with a *K*_D_ value of 6.89 µM (Figure 2). SCT-Asn-OA and SCT-Asn-GA exhibited the next best *K*_D_ values of 11.24 and 16.35 µM, respectively. The interaction between SA and HA was weak [49], indicating that oligosaccharides and pentacyclic triterpene moieties could enhance this affinity. Additionally, the affinity of the four conjugates to H5N1 was enhanced; the most active conjugate was SCT-Asn-OA, with a *K*_D_ value of 9.10 µM (Figure 2), followed by SCT-Asn-GA, with a *K*_D_ value of 13.24 µM. Although the affinity of the other two conjugates also improved, their *K*_D_ value exceeded 100 µM. Typically, the affinity of most of the conjugates to HA was higher than that of the parent oligosaccharides and pentacyclic triterpenes. Furthermore, the conjugates exhibited dose-dependent responses, which appeared not to follow specific rules. The fine structures of various pentacyclic triterpenes probably formed various bonds with HA, thus exhibiting various affinities. Notably, the tested conjugates exhibited a binding behavior distinct from that of pentacyclic triterpenes (UA, BA, OA, and GA). The pentacyclic triterpenes exhibited rapid association and dissociation with HA, whereas the conjugates exhibited slow association and dissociation with HA, as evident from the lower association rate constant (*K*_a_) and dissociation rate constant (*K*_d_) values. The interaction of conjugates with HA indicated that the conjugates could form stable complexes with HA, with low *K*_D_ values, thus improving antiviral activity against influenza virus. This result is consistent with that of a previous report [38].

## 3. Materials and Methods

### 3.1. Chemistry

ESI-MS was performed using an LCQ Fleet spectrometer (ThermoFisher Scientific, Waltham, MA, USA) in positive and negative modes. Electron ionization mass spectrometry (EI-MS) was performed with a DFS spectrometer (ThermoFisher Scientific, MA, USA) in positive mode. NMR spectra were recorded on a Bruker AVANCE III HD and AVANCE III 400 spectrometer (Bruker Daltonics, Inc., Billerica, MA, USA) at ambient temperature. ^1^H-NMR chemical shifts were referenced to the internal standard TMS (δ_H_ = 0.00). ^13^C-NMR chemical shifts were referenced to the solvent signal (δ_C_ = 77.16 for the central line of CDCl_3_, δ_C_ = 49.00 for the central line of CD_3_OD). TLC was performed on a precoated silica gel HSGF_254_ (layer thickness = 0.2 mm; Yantai Jiangyou Silica gel Development Co., Ltd., Yantai, China). Staining with an iodine and H_2_SO_4_/vanillin solution and subsequent heating were applied for detection. Analytic HPLC was performed on an Agilent 1260 II HPLC instrument (Agilent, Palo Alto, CA, USA) with a XBridge BEH HILIC column (5 µm, 4.6 mm × 250 mm, Waters, Milford, MA, USA) at 25 °C. The column was eluted with a linear gradient of 80–64% MeCN and 20–36% 15 mM KH_2_PO_4_ at 0–10 min, and then eluted with 64% MeCN and 36% 15 mM KH_2_PO_4_ for 30 min at a flow rate of 1.2 mL/min. Sephadex G-25 and Sephadex G-50 (GE Healthcare, Buckinghamshire, UK) were eluted with purified water, and Sephadex LH-20 (GE Healthcare, Buckinghamshire, UK) was eluted with methanol. C18 (Fuji Silysia Chemical Ltd, Aichi, Japan) was activated using methanol and eluted with various concentrations of methanol. Finally, the sample was lyophilized with a lyophilizer (Christ, Alpha 1-2 LD plus, Osterode, Germany).

Pronase was purchased from Roche, and all chemicals were used without further purification. The intermediates and final conjugates were obtained as follows.

#### 3.1.1. Preparation of SGP

An extraction method detailed in relevant literature [12] was used for the experiments. First, 20 kg of egg yolk powder, obtained from commercial suppliers, was defatted twice with 95% ethanol. The residue was extracted twice with 40% ethanol. After filtration, the filtrate was concentrated under a reduced pressure at 40 °C, and then 40% cold ethanol was added to precipitate the proteins, which were then removed through centrifugation. The solution was purified through active carbon/celite column chromatography, and the column was eluted with water, 5% acetonitrile, 10% acetonitrile, and 25% acetonitrile. ESI-MS revealed that SGP was in the 25% acetonitrile elution portion. Subsequently, the solution was purified through size-exclusion chromatography, and the solutions containing SGP were combined and lyophilized to obtain 10.3 g of white powdery solid (0.5 mg SGP/g egg yolk powder).

#### 3.1.2. Preparation of SCT-Asn

SGP (201.1 mg) was dissolved in a buffer solution (0.1 M Tris, 0.5% SDS, 10 mM CaCl_2_, pH 7.5). Pronase (202.7 mg) was added, and then the mixture was reacted at 37 °C for 7 days. Furthermore, 200 mg of pronase was added daily to promote the reaction. The reaction was monitored through HPLC until SGP was converted into SCT-Asn. Here, *t*_R_ (SCT-Asn) = 13.8 min and *t*_R_ (SGP) = 21.7 min. After the reaction solution was lyophilized, the residue was dissolved in a small amount of water, then purified using the Sephadex G-50 column and eluted with purified water. The product fraction was collected and lyophilized to obtain a white solid (120.4 mg, yield = 73.4%). ESI-MS (*m/z*): 1168.7 [M − 2H]^2−^, C_88_H_144_N_8_O_64_. ^1^H-NMR (400 MHz, D_2_O) δ 5.13 (s, 1H, H-1 of Man-4), 5.06 (d, *J* = 9.6 Hz, 1H, H-1 of GlcNAc-1)), 4.94 (s, 1H, H-1 of Man-4’), 4.77 (s, 1H, H-1 of Man-3), 4.61–4.59 (m, 3H, H-1 of GlcNAc-2, GlcNAc-5, GlcNAc-5’), 4.45–4.43 (m, 2H, H-1 of Gal-6, Gal-6’), 4.25 (s, 1H, H-2 of Man-3), 4.19 (s, 1H, H-2 of Man-4), 4.11 (s, 1H, H-2 of Man-4’), 3.09–2.96 (m, 2H, β-CH_2_ of Asn), 2.67–2.62 (m, 2H, H-3_eq_ of NeuAc, NeuAc’), 2.08–1.99 (m, 18H, Ac of GlcNAc-1, GlcNAc-2, GlcNAc-5, GlcNAc-5’, NeuAc, NeuAc’), 1.82 (t, *J* = 12.2 Hz, 2H, H-3_ax_ of NeuAc, NeuAc’). ^13^C-NMR (151 MHz, D_2_O) δ 177.7, 177.7, 177.6, 177.5, 176.4, 175.7, 175.3, 107.2, 106.4, 104.1, 103.3, 103.0, 103.0, 102.4, 102.2, 102.1, 99.8, 83.5, 83.4, 83.3, 82.5, 81.5, 80.9, 79.2, 79.0, 77.3, 76.5, 76.4, 76.1, 75.6, 75.4, 75.3, 75.0, 74.9, 74.8, 74.6, 73.6, 73.0, 72.3, 71.2, 71.0, 70.2, 68.7, 68.5, 66.2, 65.5, 64.5, 63.6, 63.1, 62.7, 62.2, 57.8, 57.5, 56.4, 54.7, 53.7, 42.9, 37.8, 25.3, 25.1, 24.9.

#### 3.1.3. Preparation of SCT-Asn-Alkyne

First, N-(4-pentynoyloxy)-succinimide was synthesized [41]. 4-Pentynoic acid (392.0 mg, 1 eq) and N-hydroxysuccinimide (466.0 mg, 1 eq) were dissolved in dry THF (7 mL) and cooled to 0 °C. Next, N’-dicyclohexylcarbodiimide (852.0 mg, 1 eq) dissolved in dry THF (2 mL) was added dropwise. The mixture was stirred for 1.5 h at 0 °C, slowly warmed to room temperature, and then stirred for an additional 4 h. The precipitate was filtered, and the solvent was evaporated at a reduced pressure. The residue was dissolved in ethyl acetate, washed with saturated NaHCO_3_ solution and brine, and dried over Na_2_SO_4_. The solvent was evaporated at a reduced pressure, and the residue dissolved in MeOH was subject to gel filtration with a Sephadex LH-20 column. The column was eluted with MeOH, and product fractions were combined and concentrated in vacuo to yield pent-4-ynoic acid 1-oxysuccinimidyl ester as a white powder (545.1 mg, yield = 69.9%). EI-MS (*m/z*): 195 [M]^+^, C_9_H_9_NO_4_. ^1^H-NMR (600 MHz, CD_3_OD) δ 2.87 (t, *J* = 7.2 Hz, 2H, H-4), 2.83 (s, 4H, H-7), 2.58 (td, *J* = 7.2, 2.7 Hz, 2H, H-3), 2.33 (t, *J* = 2.7 Hz, 1H, H-1). ^13^C-NMR (101 MHz, CD_3_OD) δ 171.7, 168.9, 82.2, 70.9, 31.2, 26.5, 14.7.

SCT-Asn (152.4 mg, 1 eq) and the succinimidyl ester from the previous step (64.5 mg, 5 eq) were dissolved in 4 mL of solvent (H_2_O/DMF 1/1). Triethylamine (27.2 μL, 3 eq) was slowly added to the solvent. The mixture was stirred overnight, and the residue was subjected to gel filtration with a Sephadex G-25 column. The column was eluted with H_2_O, and product fractions were combined and lyophilized to yield SCT-Asn-alkyne as a white solid (89.0 mg, yield = 56.7%). ESI-MS (*m/z*): 1209.0 [M − 2H]^2−^, C_93_H_148_N_8_O_65_. ^1^H-NMR (400 MHz, D_2_O) δ 5.13 (s, 1H, H-1 of Man-4), 5.04 (d, *J* = 9.6 Hz, 1H, H-1 of GlcNAc-1), 4.94 (s, 1H, H-1 of Man-4’), 4.77 (s, 1H, H-1 of Man-3), 4.61–4.59 (m, 3H, H-1 of GlcNAc-2, GlcNAc-5, GlcNAc-5’), 4.45–4.43 (m, 2 H, H-1 of Gal-6, Gal-6’), 4.25 (s, 1H, H-2 of Man-3), 4.19 (s, 1H, H-2 of Man-4), 4.11 (s, 1H, H-2 of Man-4’), 2.92–2.80 (m, 2H,β-CH_2_ of Asn), 2.67–2.62 (m, 2H, H-3_eq_ of NeuAc, NeuAc’), 2.49-2.48 (m, 4H, CH_2_ CH_2_ -alkyne), 2.36 (t, *J* = 2.3 Hz, 1H, H of alkyne), 2.07-1.99 (m, 18H, Ac of GlcNAc-1, GlcNAc-2, GlcNAc-5, GlcNAc-5’, NeuAc, NeuAc’), 1.82 (t, *J* = 12.2 Hz, 2H, H-3_ax_ of NeuAc, NeuAc’). ^13^C-NMR (151 MHz, D_2_O) δ 177.8, 177.7, 177.6, 177.4, 177.0, 175.6, 174.5, 166.0, 165.8, 120.3, 118.3, 106.6, 104.3, 103.4, 102.5, 102.2, 101.9, 99.9, 98.2, 86.2, 83.6, 83.4, 82.6, 81.6, 81.2, 79.4, 79.2, 78.3, 77.4, 76.5, 75.8, 75.7, 75.5, 75.4, 75.0, 75.0, 74.0, 73.6, 73.4, 73.2, 73.1, 72.4, 71.4, 71.3, 71.2, 70.5, 70.3, 69.7, 68.7, 66.3, 66.1, 65.9, 64.7, 64.6, 64.0, 63.2, 62.9, 62.8, 57.9, 57.6, 56.8, 55.0, 54.7, 51.9, 42.1, 41.8, 39.8, 37.0, 25.4, 25.4, 25.3, 25.2, 25.1, 25.0, 25.0, 17.3.

#### 3.1.4. General Procedure for Preparing Pentacyclic Triterpenes with the Azide Group

K_2_CO_3_ (2 eq) was added to a solution of pentacyclic triterpenes (1.1 eq) and Bromo-PEG5-azide (1 eq) in DMF. The reaction mixture was stirred at room temperature for 18–24 h and concentrated in vacuo. The residue was diluted with dichloromethane or ethyl acetate, and the extract was washed successively with saturated NaHCO_3_ and brine before being dried with Na_2_SO_4_, filtered, and concentrated *in vacuo*. The residue was purified through gel filtration with a Sephadex LH-20 column, and the column was eluted with MeOH; product fractions were combined and concentrated *in vacuo*.

##### UA-N_3_

UA-N_3_ was prepared from ursolic acid (201.1 mg, 1.1 eq) and Bromo-PEG5-azide (147.9 mg, 1 eq) according to the general procedure. The residue was purified to yield UA-N_3_ as a colorless oil (201.5 mg, yield = 67.6%). ESI-MS (*m/z*): 747.2 [M + H]^+^, C_42_H_71_N_3_O_8_. ^1^H-NMR (600 MHz, CDCl_3_) δ 5.24 (t, *J* = 3.7 Hz,1H, CH-12), 4.15 (t, *J* = 5.1 Hz, 2H), 3.69–3.63 (m, 22H), 3.39 (t, *J* = 5.1 Hz, 2H), 3.22 (dd, *J* = 11.3, 4.7 Hz, 1H, CH-3), 2.24 (d, *J* = 11.3 Hz, 1H, CH-18), 2.00 (td, *J* = 13.5, 4.5 Hz, 1H, CH_2(Hβ)_-15), 1.91–1.89 (m, 2H), 1.81–1.76 (m, 1H), 1.74 (s, 2H), 1.70–1.65 (m, 3H), 1.63–1.56 (m, 4H), 1.54–1.46 (m, 5H), 1.36–1.26 (m, 6H), 1.08 (s, 3H, CH_3_-27), 1.06–1.05 (m, 1H, CH-20), 0.99 (s, 3H, CH_3_-23), 0.95 (d, *J* = 6.3 Hz, 3H, CH_3_-29), 0.92 (s, 3H, CH_3_-26), 0.86 (d, *J* = 6.5 Hz, 3H, CH_3_-30), 0.78 (s, 3H, CH_3_-24), 0.75 (s, 3H, CH_3_-25), 0.72 (d, *J* = 11.8 Hz, 1H, CH-5). ^13^C-NMR (151 MHz, CDCl_3_) δ 177.6, 138.2, 125.7, 79.2, 70.8, 70.8, 70.8, 70.8, 70.7, 70.7, 70.7, 70.2, 69.3, 63.4, 55.3, 53.0, 50.8, 48.2, 47.7, 42.2, 39.7, 39.2, 39.0, 38.9, 38.8, 37.1, 36.7, 33.2, 30.8, 28.3, 28.1, 27.4, 24.3, 23.7, 23.4, 21.3, 18.5, 17.2, 17.1, 15.8, 15.6.

##### OA-N_3_

OA-N_3_ was prepared from oleanolic acid (50.3 mg, 1.1 eq) and Bromo-PEG5-azide (37.4 mg, 1 eq) according to the general procedure. The residue was purified to yield OA-N_3_ as a colorless oil (51.8 mg, yield = 68.7%). ESI-MS (*m/z*): 746.5 [M + H]^+^, C_42_H_71_N_3_O_8_. ^1^H-NMR (600 MHz, CDCl_3_) δ 5.28 (t, *J* = 3.7 Hz, 1H, CH-12), 4.22–4.13 (m, 2H), 3.68–3.64 (m, 22H), 3.39 (t, *J* = 5.1 Hz, 2H), 3.22–3.19 (m, 1H, CH-3), 2.86 (dd, *J* = 13.9, 4.6 Hz, 1H, CH-18), 1.99–1.94 (m, 2H), 1.89–1.86 (m, 2H, CH_2_-22), 1.72–1.69 (m, 1H), 1.67–1.59 (m, 7H), 1.55–1.52 (m, 4H), 1.47-1.25 (m, 6H), 1.20–1.15 (m, 3H), 1.13 (s, 3H, CH_3_-27), 1.06 (m, 2H, CH_2_-1), 0.98 (s, 3H, CH_3_-25), 0.92 (s, 3H, CH_3_-23), 0.90 (s,3H, CH_3_-30), 0.90 (s, 3H, CH_3_-29), 0.78 (s, 3H, CH_3_-26), 0.73 (s, 3H, CH_3_-24), 0.72 (m, 1H, CH-5). ^13^C-NMR (101 MHz, CDCl_3_) δ 177.6, 143.7, 122.4, 78.9, 70.7, 70.6, 70.6, 70.6, 70.5, 70.5, 70.5, 70.0, 69.1, 63.3, 55.2, 50.6, 47.6, 46.6, 45.8, 41.7, 41.2, 39.3, 38.7, 38.4, 37.0, 33.8, 33.1, 32.7, 32.3, 30.7, 28.1, 27.6, 27.2, 25.8, 23.6, 23.4, 22.9, 18.3, 17.0, 15.6, 15.3.

##### BA-N_3_

BA-N_3_ was prepared from betulinic acid (50.3 mg, 1.1 eq) and Bromo-PEG5-azide (40.9 mg, 1 eq) according to the general procedure. The residue was purified to yield BA-N_3_ as a colorless oil (56.8 mg, yield = 69.0%). ESI-MS (*m/z*): 746.3 [M + H]^+^, C_42_H_71_N_3_O_8_. ^1^H-NMR (400 MHz, CD_3_OD) δ 4.72 (d, *J* = 2.3 Hz, 1H, Ha-29), 4.60 (d, *J* = 2.4 Hz, 1H, Hb-29), 4.28–4.17 (m, 2H), 3.71–3.63 (m, 22H), 3.37 (t, *J* = 4.9 Hz, 2H), 3.34 (s, 1H), 3.12 (dd, *J* = 11.1, 5.1 Hz, 1H, CH-3), 3.02 (td, *J* = 10.5, 4.5 Hz, 1H, H-19), 2.29–2.22 (m, 2H), 1.94–1.87 (m, 2H, CH_2_-16), 1.70 (s, 3H, CH_3_-30), 1.65–1.52 (m, 4H), 1.49–1.35 (m, 9H), 1.32–1.23 (m, 2H), 1.19–1.04 (m, 2H), 1.00 (s, 3H, CH_3_-26), 0.95 (s, 6H, CH_3_-23, 24), 0.86 (s, 3H, CH_3_-25), 0.75 (s, 3H, CH_3_-27), 0.72–0.69 (m, 1H). ^13^C-NMR (101 MHz, CD_3_OD) δ 177.4, 151.8, 110.4, 79.6, 71.6, 71.6, 71.6, 71.6, 71.5, 71.2, 70.3, 64.2, 57.9, 56.8, 51.9, 51.8, 50.6, 49.4, 48.5, 43.6, 42.0, 40.1, 40.0, 39.6, 38.3, 38.0, 35.6, 33.1, 31.7, 30.8, 28.7, 28.0, 26.8, 22.1, 19.6, 19.5, 16.8, 16.8, 16.2, 15.2.

##### GA-N_3_

GA-N_3_ was prepared from glycyrrhetinic acid (52.2 mg, 1.1 eq) and Bromo-PEG5-azide (37.1 mg, 1 eq) according to the general procedure. The residue was purified to yield GA-N_3_ as a colorless oil (58.2 mg, yield = 76.6%). ESI-MS (*m/z*): 760.8 [M + H]^+^, C_42_H_69_N_3_O_9_. ^1^H-NMR (400 MHz, CD_3_OD) δ 5.62 (s, 1H, CH-12), 4.38–4.18 (m, 2H), 4.10 (q, *J* = 7.1 Hz, 1H), 3.74–3.62 (m, 22H), 3.41–3.39 (m, 2H), 3.17 (m, 1H, CH-3), 2.73 (dt, *J* = 13.4, 3.6 Hz, 1H), 2.44 (s, 1H), 2.21–2.11 (m, 2H), 1.78–1.46 (m, 8H), 1.42 (s, 3H, CH_3_-27), 1.28–1.22 (m, 3H), 1.16 (s, 3H, CH_3_-29), 1.14 (s, 6H, CH_3_-26, CH_3_-25), 1.06–1.01 (m, 2H), 0.99 (s, 3H, CH_3_-23), 0.83 (s, 3H, CH_3_-24), 0.80 (s, 3H, CH_3_-28). ^13^C-NMR (101 MHz, CD_3_OD) δ 202.3, 178.1, 172.5, 129.0, 79.3, 71.4, 71.4, 71.4, 71.3, 71.0, 70.3, 64.6, 63.1, 61.5, 56.1, 51.8, 49.8, 49.7, 46.7, 45.3, 44.5, 42.3, 40.3, 40.2, 38.9, 38.3, 33.8, 32.9, 32.0, 29.2, 28.7, 28.6, 27.8, 27.6, 27.4, 23.9, 20.9, 19.3, 18.6, 17.0, 16.4, 14.5.

#### 3.1.5. General Procedure for Preparing SCT-Asn-Pentacyclic Triterpene Conjugates

In this procedure, TBTA, 0.1 M CuSO_4_ and 0.1 M sodium ascorbate was added to a solution of SCT-Asn-alkyne and pentacyclic triterpenoids in a solvent of THF/H_2_O (3/2 *v/v*). The solution was stirred at room temperature for 5 h. The solvent was concentrated, then the residue was redissolved in MeOH and subjected to gel filtration with a Sephadex LH-20 column. The column was eluted with MeOH, and product fractions were combined. The residue was purified using a C18 column and eluted with H_2_O, 10% MeOH, 20% MeOH, 30% MeOH, 35% MeOH, 40% MeOH, 45% MeOH, 50% MeOH, 55% MeOH, 60% MeOH, 65% MeOH, 70% MeOH, 80% MeOH, 90% MeOH, and 100% MeOH, and the product fractions were combined and concentrated *in vacuo* and then lyophilized.

##### SCT-Asn-UA

SCT-Asn-UA was prepared from SCT-Asn-alkyne (50.2 mg, 1 eq) and UA-N_3_ (63.6 mg, 4 eq) according to the general procedure. SCT-Asn-UA was synthesized as a white solid (19.4 mg, yield = 29.5%). ESI-MS (negative mode, *m/z*): 1581.5 [M − 2H]^2−^, 1054.9 [M − 3H]^3−^; HR-ESI-MS (positive mode, *m/z*) 1582.1941 [M + 2H]^2+^ (calcd 1582.1954); C_135_H_219_N_11_O_73_. ^1^H-NMR (400 MHz, CD_3_OD) δ 5.24 (m, 1H, CH-12), 5.12 (s, 1H, H-1 of Man-4), 4.34–4.32 (m, 2H, H-1 of Gal-6,Gal-6’), 4.18 (s, 1H, H-2 of Man-4), 3.15 (dd, *J* = 11.0, 4.6 Hz, 1H, CH-3), 3.03–2.97 (m, 2H, β-CH_2_ of Asn), 2.78–2.75 (m, 2H, H-3_eq_ of NeuAc, NeuAc’), 2.24 (d, *J* = 11.4 Hz, 1H, CH-18), 2.10–1.84 (m, 18H, NAc of GlcNAc-1, GlcNAc-2, GlcNAc-5, GlcNAc-5’, NeuAc, NeuAc’), 1.11 (s, 3H, CH_3_-27), 0.97–0.95 (m, 9H, CH_3_-23, CH_3_-29, CH_3_-26), 0.88 (d, *J* = 6.4 Hz, 3H, CH_3_-30), 0.78–0.77 (2s, 6H, CH_3_-24, CH_3_-25).

##### SCT-Asn-OA

SCT-Asn-OA was prepared from SCT-Asn-alkyne (67.6 mg, 1 eq) and OA-N_3_ (51.8 mg, 2 eq) according to the general procedure. SCT-Asn-OA was synthesized as a white solid (13.2 mg, yield = 14.9%). C_135_H_219_N_11_O_73_, ESI-MS (negative mode, *m/z*): 1581.4 [M − 2H]^2−^, 1054.3 [M − 3H]^3−^; HR-ESI-MS (positive mode, *m/z*) 1582.1963 [M + 2H]^2+^ (calcd 1582.1954,); ^1^H-NMR (600 MHz, CD_3_OD) δ 5.25 (m, 1H, CH-12), 5.12 (s, 1H, H-1 of Man-4), 4.34–4.33 (m, 2H, H-1 of Gal-6,Gal-6’), 4.18 (s, 1H, H-2 of Man-4), 3.64–3.59 (m, 22H), 3.00 (m, 2H, β-CH_2_ of Asn), 2.89–2.87 (m, 1H, CH-18), 2.60 (m, 2H, H-3_eq_ of NeuAc, NeuAc’), 2.05–1.89 (m, 18H, NAc of GlcNAc-1, GlcNAc-2, GlcNAc-5, GlcNAc-5’, NeuAc, NeuAc’), 1.15 (s, 3H, CH_3_-27), 0.97 (s, 3H, CH_3_-25), 0.94–0.89 (m, 9H, CH_3_-23, CH_3_-30, CH_3_-29), 0.77–0.76 (2s, 6H, CH_3_-26, CH_3_-24).

##### SCT-Asn-BA

SCT-Asn-BA was prepared from SCT-Asn-alkyne (68.0 mg, 1 eq) and BA-N_3_ (56.8 mg, 2.7 eq) according to the general procedure. SCT-Asn-BA was synthesized as a white solid (21.2 mg, 23.8%). C_135_H_219_N_11_O_73_, ESI-MS (negative mode, *m/z*): 1581.3 [M − 2H]^2−^, 1054.1 [M-3H]^3^; HR-ESI-MS (positive mode, *m/z*) 1582.1974 [M + 2H]^2+^ (calcd 1582.1954); ^1^H-NMR (600 MHz, CD_3_OD) δ 5.13 (s, 1H, H-1 of Man-4), 4.34–4.33 (m, 2H, H-1 of Gal-6, Gal-6’), 3.12 (dd, *J* = 11.5, 4.7 Hz, 1H, CH-3), 2.78–2.76 (m, 2H, β-CH_2_ of Asn), 2.61–2.58 (m, 2H, H-3_eq_ of NeuAc, NeuAc’), 2.05–1.96 (m, 18H, NAc of GlcNAc-1, GlcNAc-2, GlcNAc-5, GlcNAc-5’, NeuAc, NeuAc’), 1.69 (s, 3H, CH_3_-30), 1.00 (s, 3H, CH_3_-26), 0.94 (s, 6H, CH_3_-23, 24), 0.85 (s, 3H, CH_3_-25), 0.75 (s, 3H, CH_3_-27).

##### SCT-Asn-GA

SCT-Asn-GA was prepared from SCT-Asn-alkyne (50.4 mg, 1 eq) and GA-N_3_ (58.2 mg, 3.7 eq) according to the general procedure. SCT-Asn-GA was synthesized as a white solid (11.6 mg, 17.5%). C_135_H_217_N_11_O_74_, ESI-MS (negative mode, *m/z*): 1588.2 [M − 2H]^2−^, 1058.5 [M − 3H]^3−^; HR-ESI-MS (positive mode, *m/z*) 1589.1852 [M + 2H]^2+^ (calcd 1589.1850); ^1^H-NMR (600 MHz, CD_3_OD) δ 5.61 (s, 1H, CH-12), 5.13 (s, 1H, H-1 of Man-4), 4.34–4.33 (m, 2H, H-1 of Gal-6,Gal-6’), 3.00 (m, 2H, β-CH_2_ of Asn), 2.60 (m, 2H, H-3_eq_ of NeuAc, NeuAc’), 1.42 (s, 3H, CH_3_-27), 1.16 (s, 3H, CH_3_-29), 1.14 (s, 6H, CH_3_-26, 25), 0.99 (s, 3H, CH_3_-23), 0.83 (s, 3H, CH_3_-24), 0.80 (s, 3H, CH_3_-28).

### 3.2. SPR Assay

Biacore T200 instruments (GE Healthcare) were used to evaluate the binding affinity of compounds with H1N1 (A/WSN/1933) and H5N1 (A/Hong Kong/483/97), purchased from Sino Biological Inc. (Beijing, China) through SPR, as previously described [50]. In brief, HA protein was immobilized on the surface of a CM5 chip by using the amine coupling approach at a flow rate of 10 μL/min in 10 mM sodium acetate buffer (pH 5.0). The sensor surface was activated with a 7 min injection of a mixture of 50 mM N-hydroxysuccinimide (NHS) and 200 mM 1-ethyl-3-(3-dimethylaminopropyl) carbodiimide (EDC). Next, 25 μg/mL HA protein was injected to reach the target level of approximately 15,000 RU for 420 s, and the surface was blocked with 1 M ethanolamine, pH 8.5. The coupling amount of H1N1 was 15886.4 RU, and the coupling amount of H5N1 was 7203.8 RU. Series concentrations of compounds were injected into the flow system and analyzed for 90 s, and dissociation occurred for 90 s. All binding analyses were performed in phosphate-buffered saline (PBS) with 0.05% (*v/v*) Tween 20 and 5% DMSO, pH 7.4, at 25 °C. Prior to analysis, double reference subtraction and solvent correction were performed to eliminate bulk refractive index changes, injection noise, and data drift. A Langmuir 1:1 binding model was fit using Biacore evaluation software (GE Healthcare) to determine the binding affinity.

## 4. Conclusions

A total of four SCT-Asn-pentacyclic triterpene conjugates were synthesized, and their anti-influenza activity was preliminarily investigated through SPR assays. The most promising result was observed for SCT-Asn-BA, which bonded to H1N1 with a *K*_D_ value of 6.89 µM. Furthermore, SCT-Asn-OA bonded to H5N1 with a *K*_D_ value of 9.10 µM, thus disrupting the interaction of HA with SA. Future studies should analyze the anti-influenza virus activity of these conjugates at the cellular level. Compared with the parent SCT-Asn and the pentacyclic triterpenes, the conjugate exhibited an increased affinity, which indicated that the influence of the nonsialic acid part on the structure should be considered. Generally, molecules with high affinity to HA also have the potential to inhibit influenza viruses by disrupting the attachment of viruses to host cells. This study may provide guidance for the design of anti-influenza compounds, that is, to synthesize two low-activity compounds into a conjugate.

## Figures and Tables

**Table 1:** The thermodynamic dissociation constant *K*_D_ (µM) values of compounds with H1N1(A/WSN/1933) and H5N1(A/Hong Kong/483/97) proteins.

Compounds	H1N1(A/WSN/1933)	H5N1(A/Hong Kong/483/97)
**SGP**	ND	ND
**SCT-Asn**	29.04	75.46
**UA**	136.70	ND
**BA**	ND	ND
**OA**	31.14	47.78
**GA**	584.00	>1000
**SCT-Asn-UA**	289.40	852.70
**SCT-Asn-BA**	6.89	251.60
**SCT-Asn-OA**	11.24	9.10
**SCT-Asn-GA**	16.35	13.24

ND: Not detected. SGP: Sialylglycopeptide SCT-Asn: Asparagine (Asn)-linked N-glycans. UA: Ursolic acid BA: betulinic acid OA: oleanolic acid GA: glycyrrhetinic acid.

## Data Availability

The data presented in this study are available within the article and in Supplementary Materials.

## Supplementary Materials

The following are available online. Figure S1: Chemical structures of four pentacyclic triterpene-N3. Figure S2: Chemical structures of four SCT-Asn-pentacyclic triterpene. Figure S3: SPR assay to determine the affinity of compounds to H1N1 (A/WSN/1933) protein immobilized on a CM5 sensor chip. Figure S4: SPR assay to determine the affinity of compounds to H5N1 (A/Hong Kong/483/97) protein immobilized on a CM5 sensor chip. ^1^H-, ^13^C-NMR, and MS spectra of intermediate and final products. High resolution mass spectra of four conjugates.
