# Supplementary material for: Synthesis of Four Pentacyclic Triterpene–Sialylglycopeptide Conjugates and Their Affinity Assays with Hemagglutinin
Source: Molecules. 2021 Feb 8;26(4):895. doi: 10.3390/molecules26040895 (PMC7915185; doi:10.3390/molecules26040895)
Supplement: Supplementary file 1 [file molecules-26-00895-s001.pdf]

Supporting Information

Synthesis of four pentacyclic triterpene–sialylglycopeptide conjugates and their affinity assays with hemagglutinin

Mei Luo, Ximin Wu, Yiming Li * and Fujiang Guo *

School of Pharmacy, Shanghai University of Traditional Chinese Medicine, Shanghai 201203, China
* Correspondence: yminlius@163.com; gfj@shutcm.edu.cn; Tel.: +86-215-132-2181

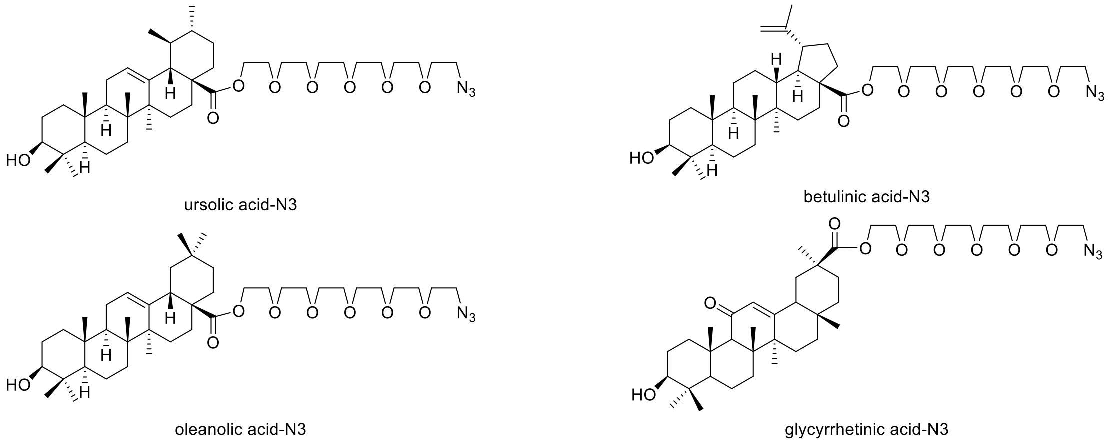

Figure S1. Chemical structures of four pentacyclic triterpene-N_3.

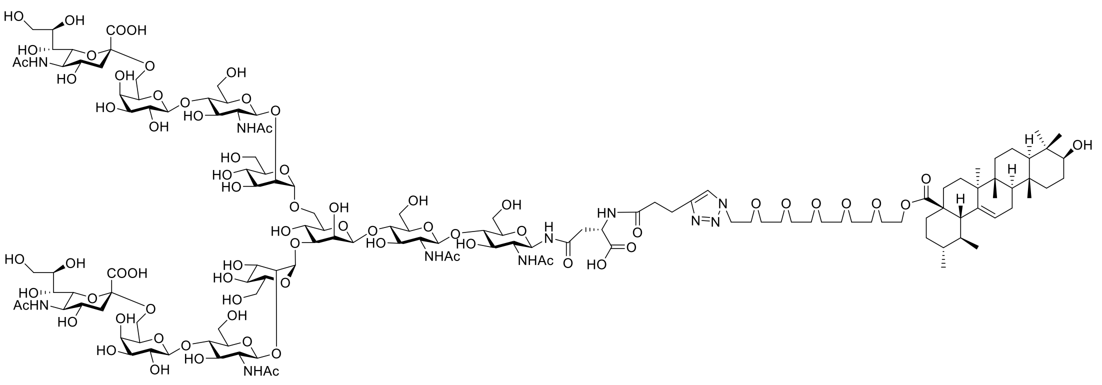

SCT-Asn-ursolic acid

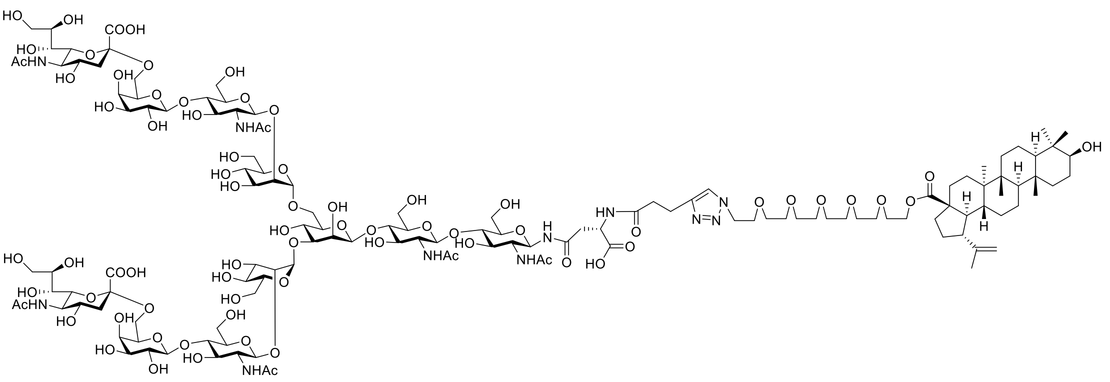

SCT-Asn-betulinic acid

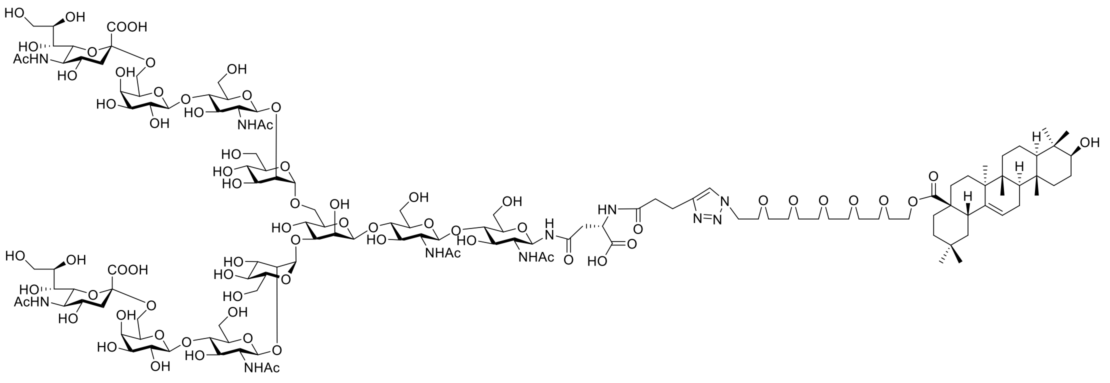

SCT-Asn-oleanolic acid

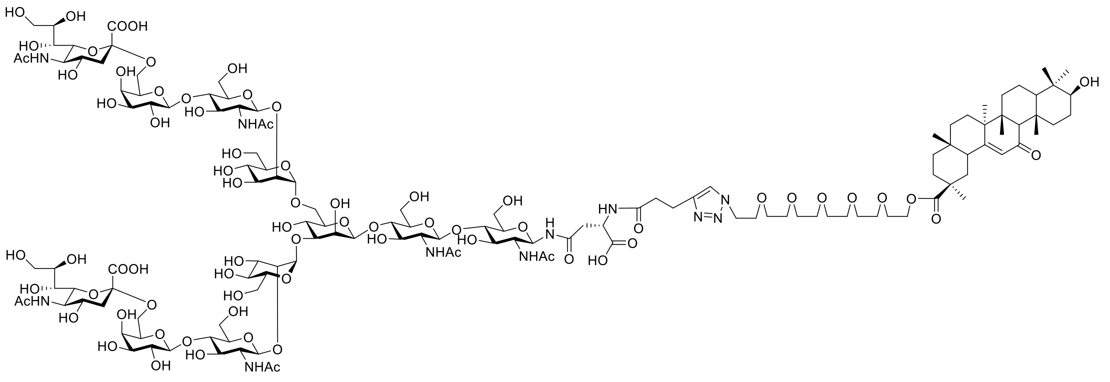

SCT-Asn-glycyrrhetinic acid

Figure S2. Chemical structures of four SCT-Asn-pentacyclic triterpene.

K_D(SGP): ND

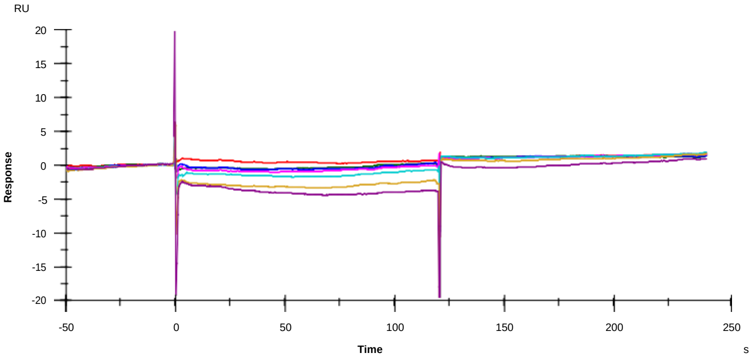

K_D(SCT-Asn): 29.04 μM

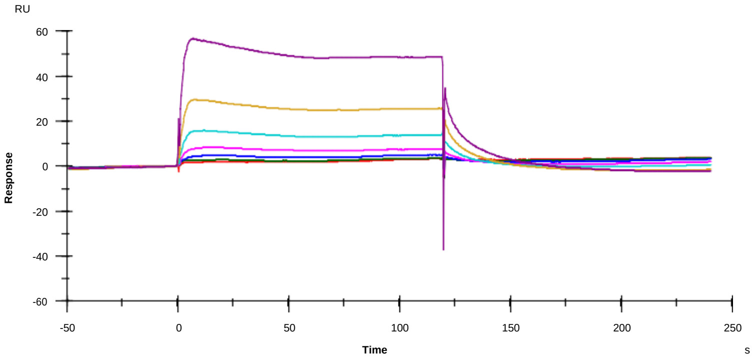

K_D(UA): 136.70 μM

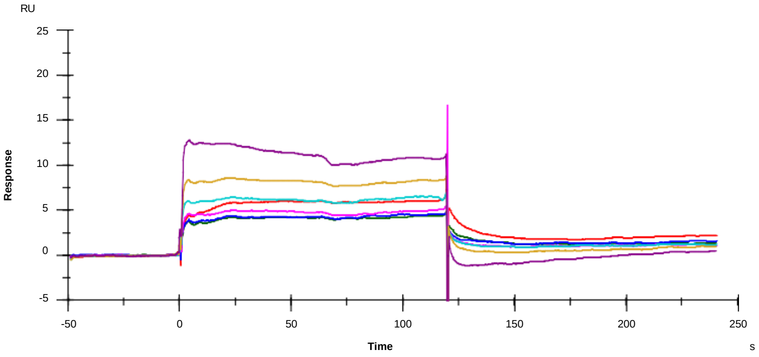

K_D(BA): ND

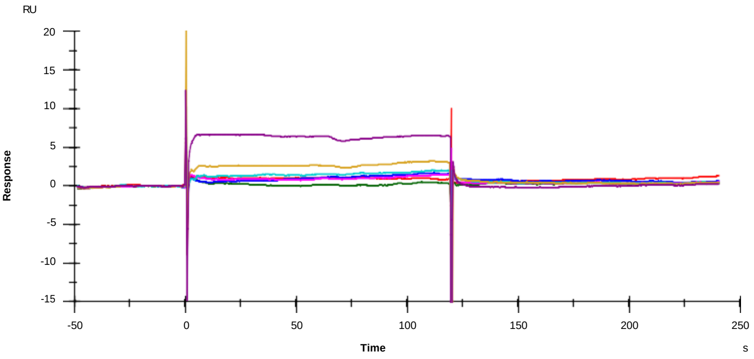

K_D(OA): 31.14 μM

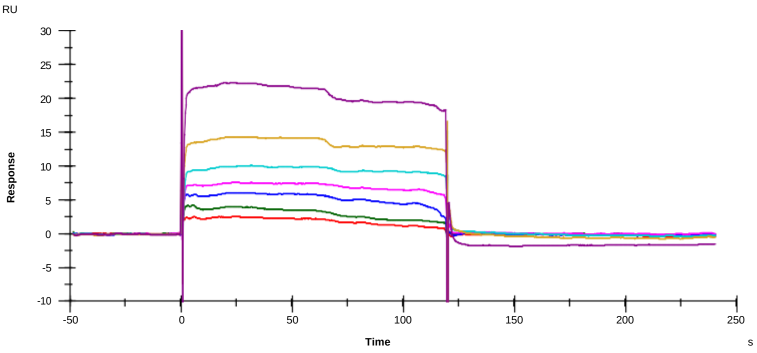

K_D(GA): 584.00 μM

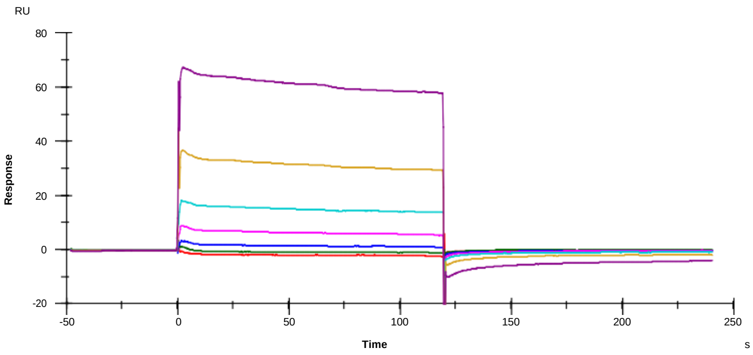

K_D(SCT-Asn-UA): 289.40 μM

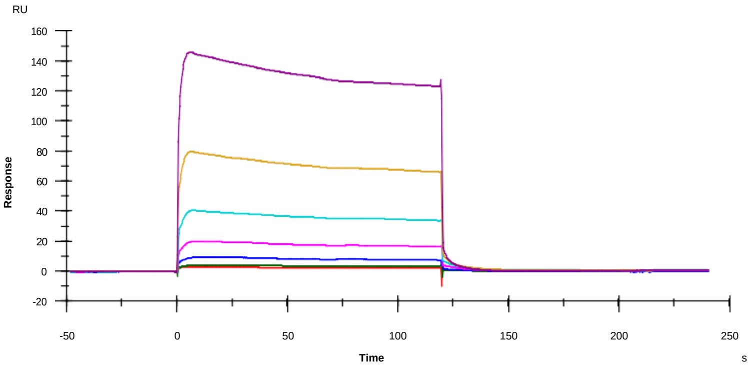

K_D(SCT-Asn-BA): 6.89 μM

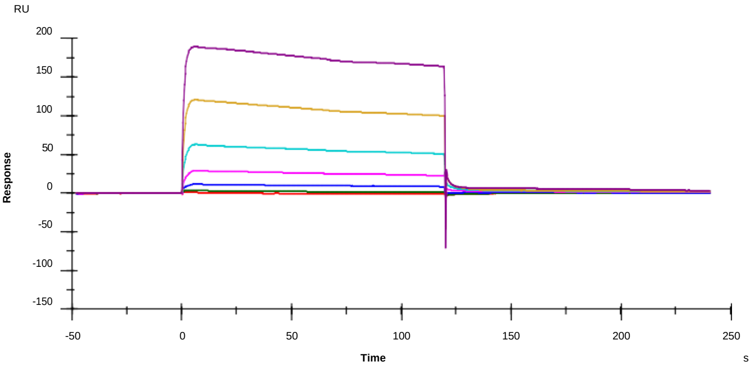

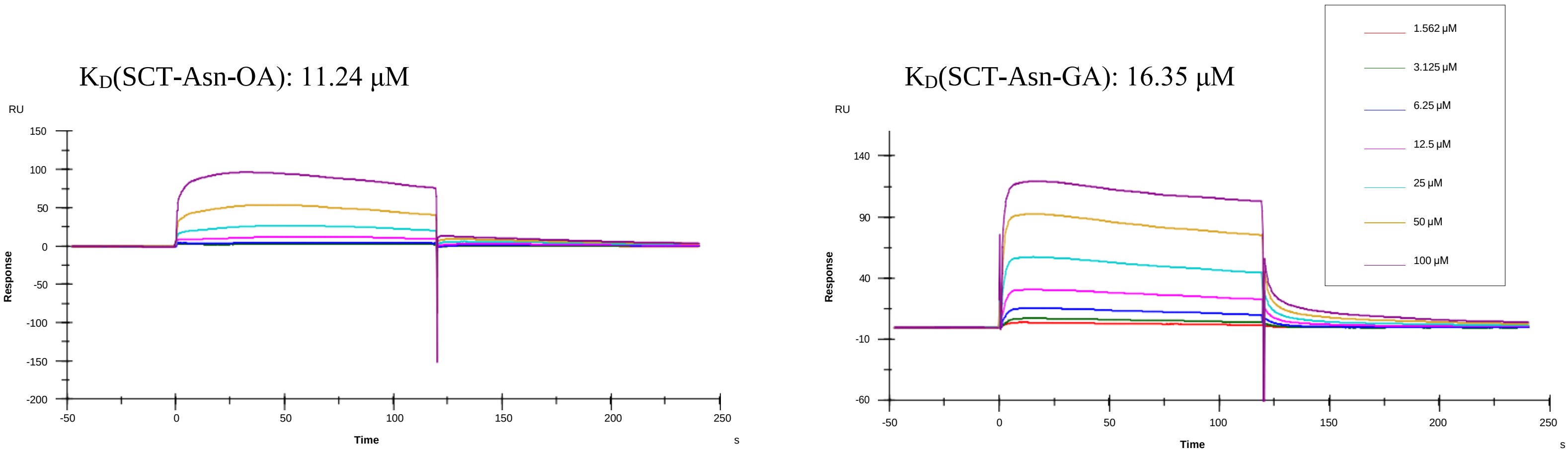

Figure S3. SPR assay to determine the affinity of compounds to H1N1 (A/WSN/1933) protein immobilized on a CM5 sensor chip.

K_D(SGP): ND

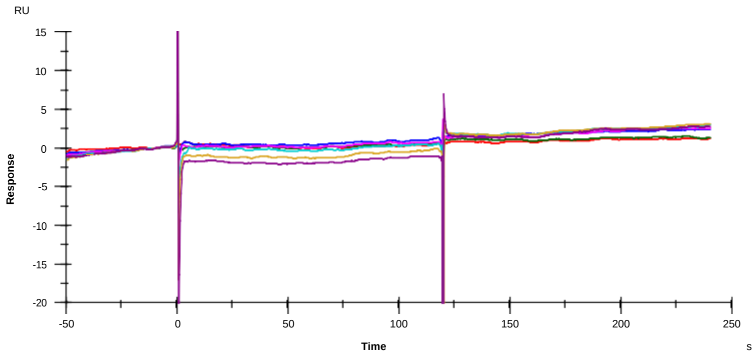

K_D(SCT-Asn): 75.46 μM

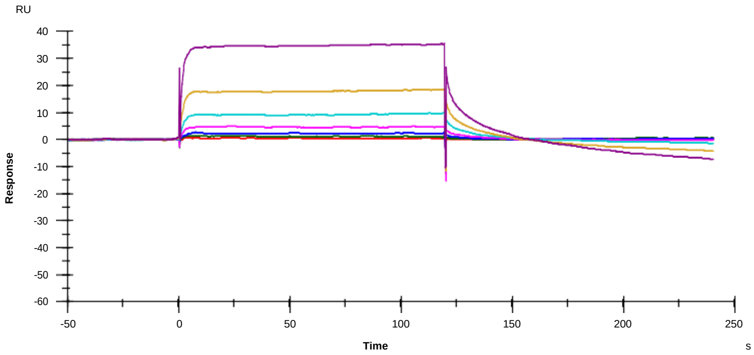

K_D(UA): ND

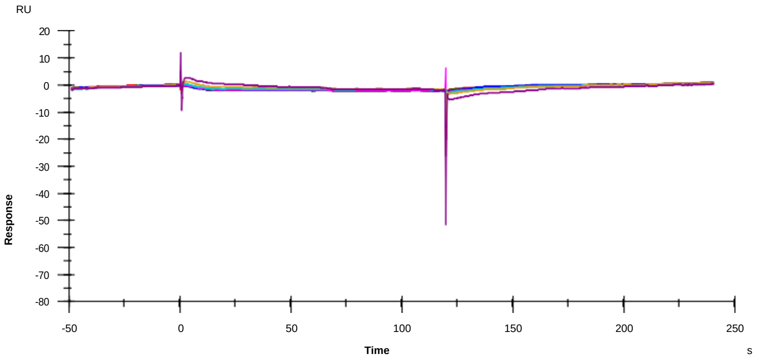

K_D(BA): ND

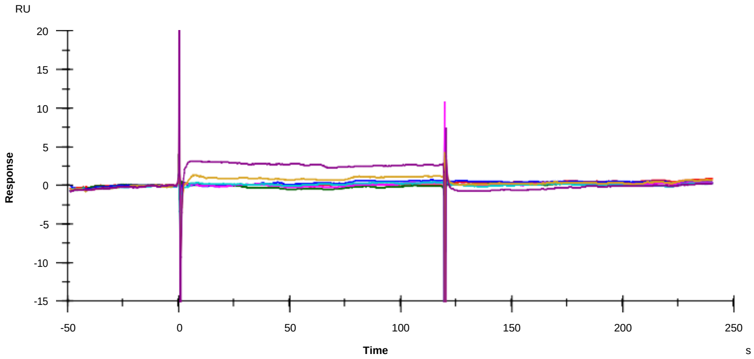

K_D(OA): 47.78 μM

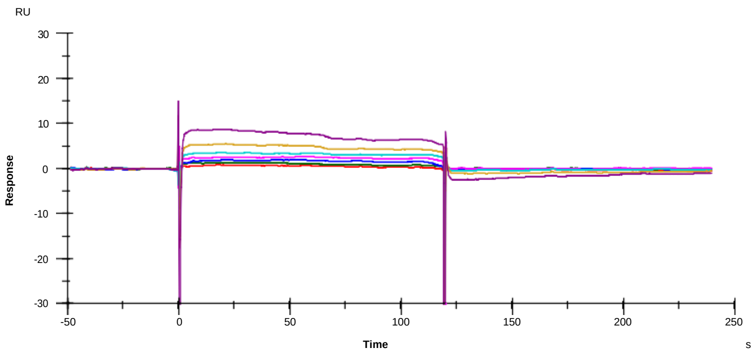

K_D(GA): 2280 μM

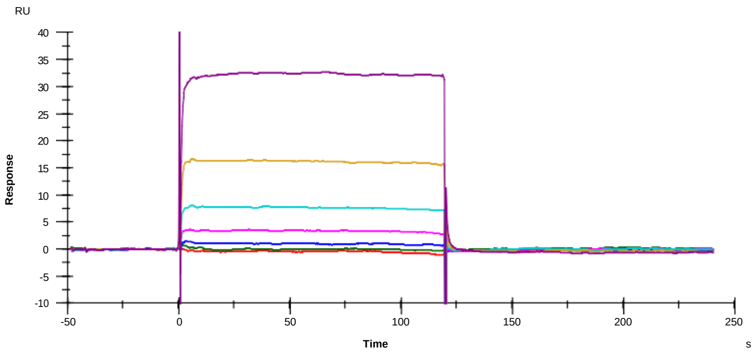

K_D(SCT-Asn-UA): 852.70 μM

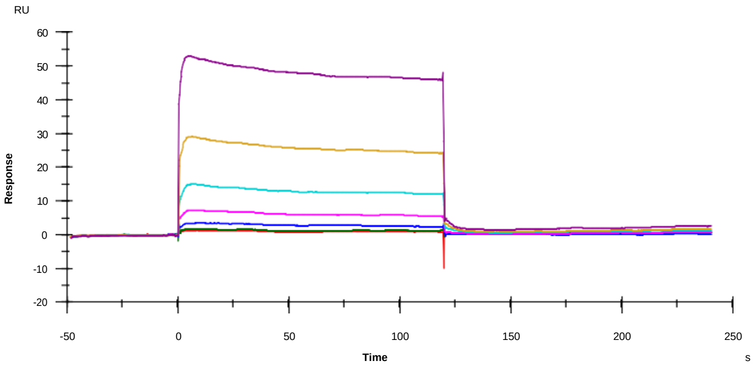

K_D(SCT-Asn-BA): 251.60 μM

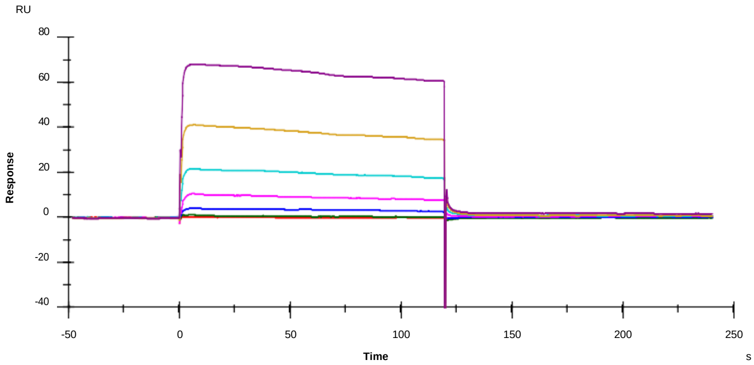

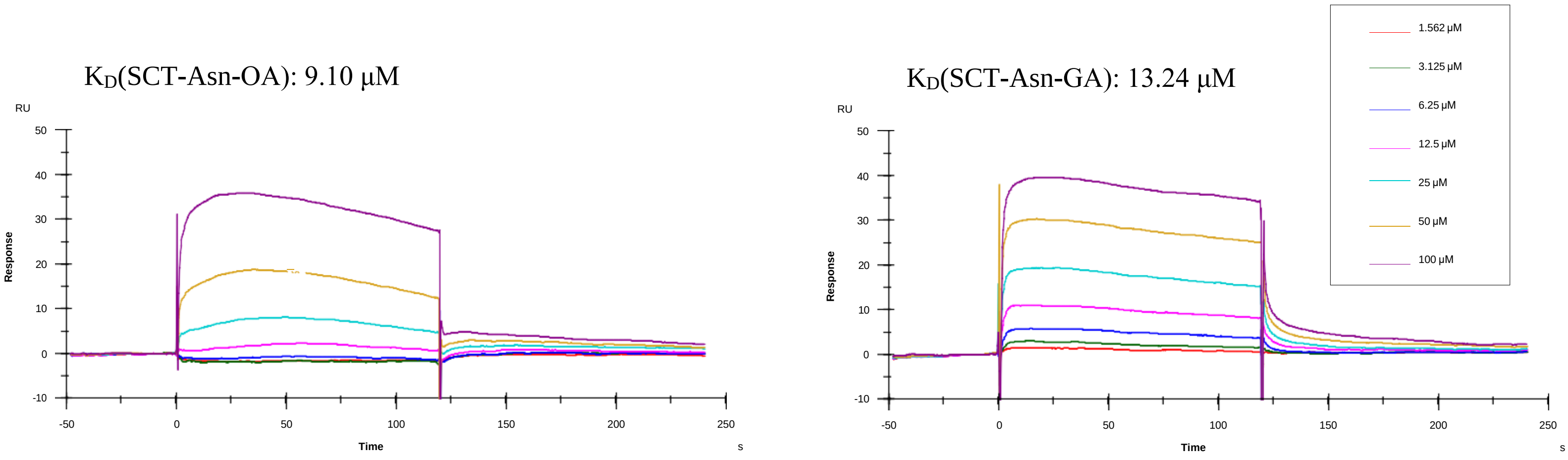

Figure S4. SPR assay to determine the affinity of compounds to H5N1 (A/Hong Kong/483/97) protein immobilized on a CM5 sensor chip.

^{1}H-, ^{13}C-NMR, and MS spectra of intermediate and final products.

1. ^{1}H NMR (600 MHz, CD_3OD) spectrum of pent-4-ynoic acid succinimidyl ester

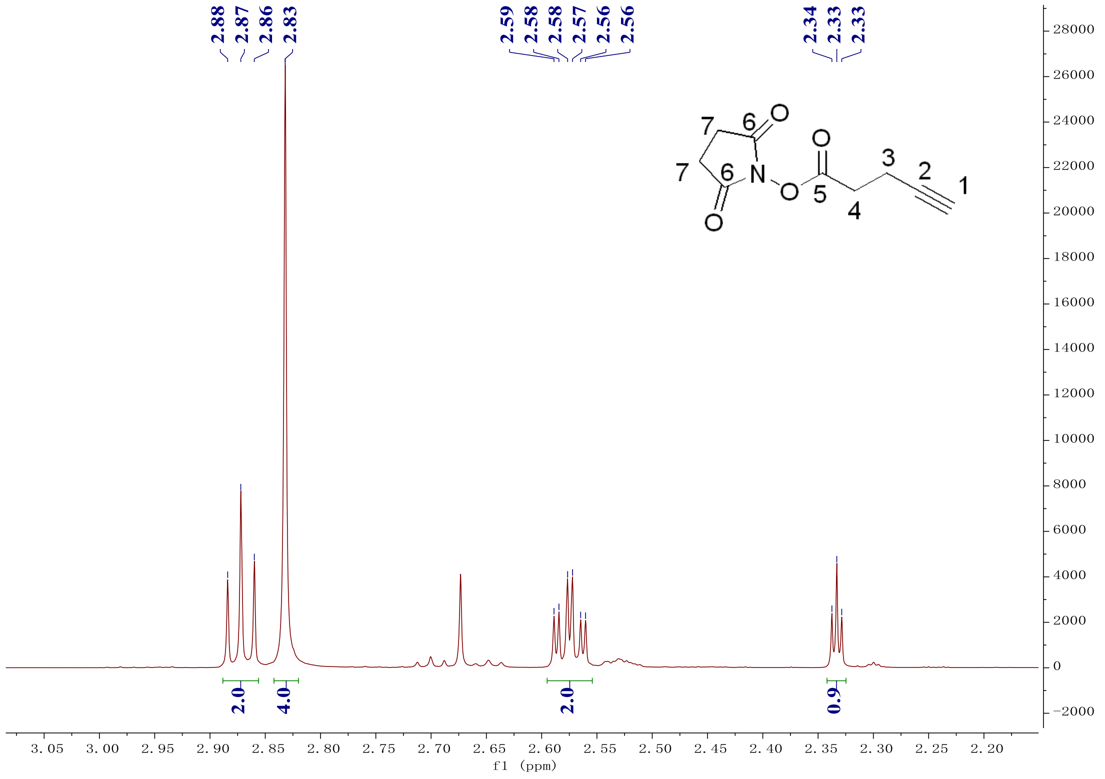

2. ^{13}C NMR (101 MHz, CD_3OD) spectrum of pent-4-ynoic acid succinimidyl ester

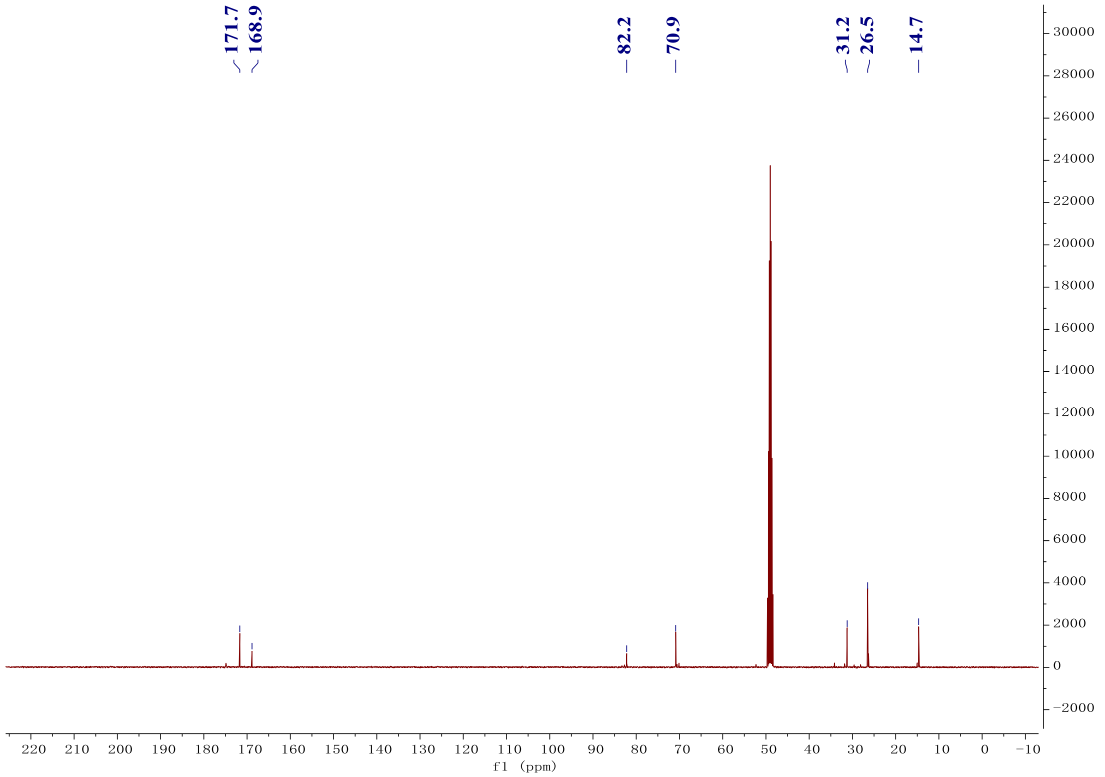

3. EI-MS spectrum of pent-4-ynoic acid succinimidyl ester

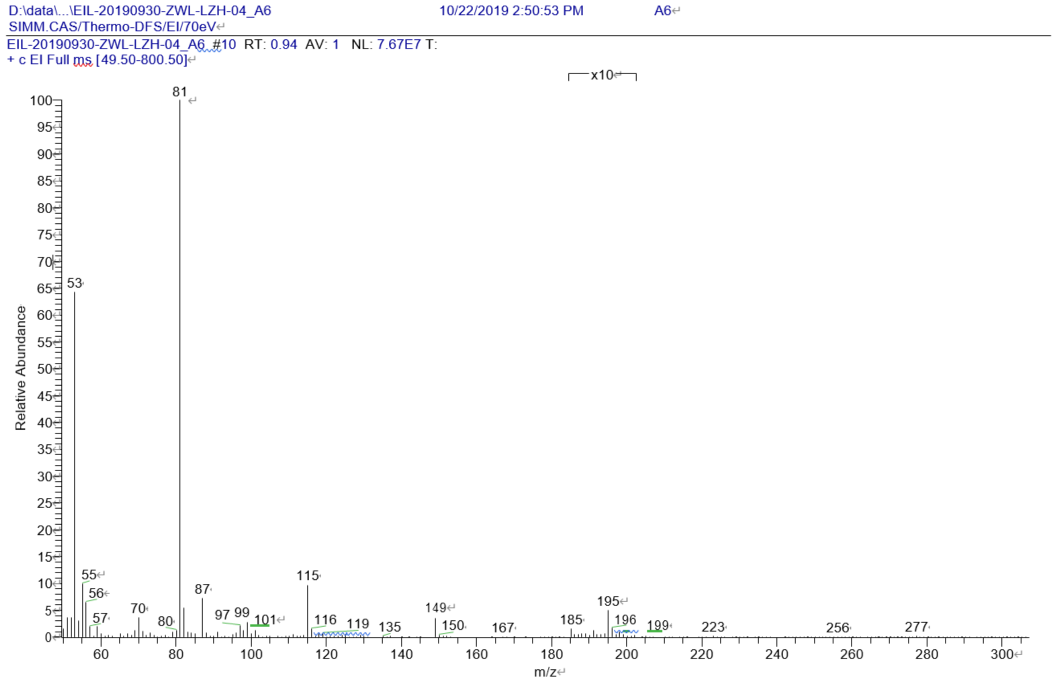

4. ¹H NMR (400 MHz, D_2O) spectrum of SCT-Asn

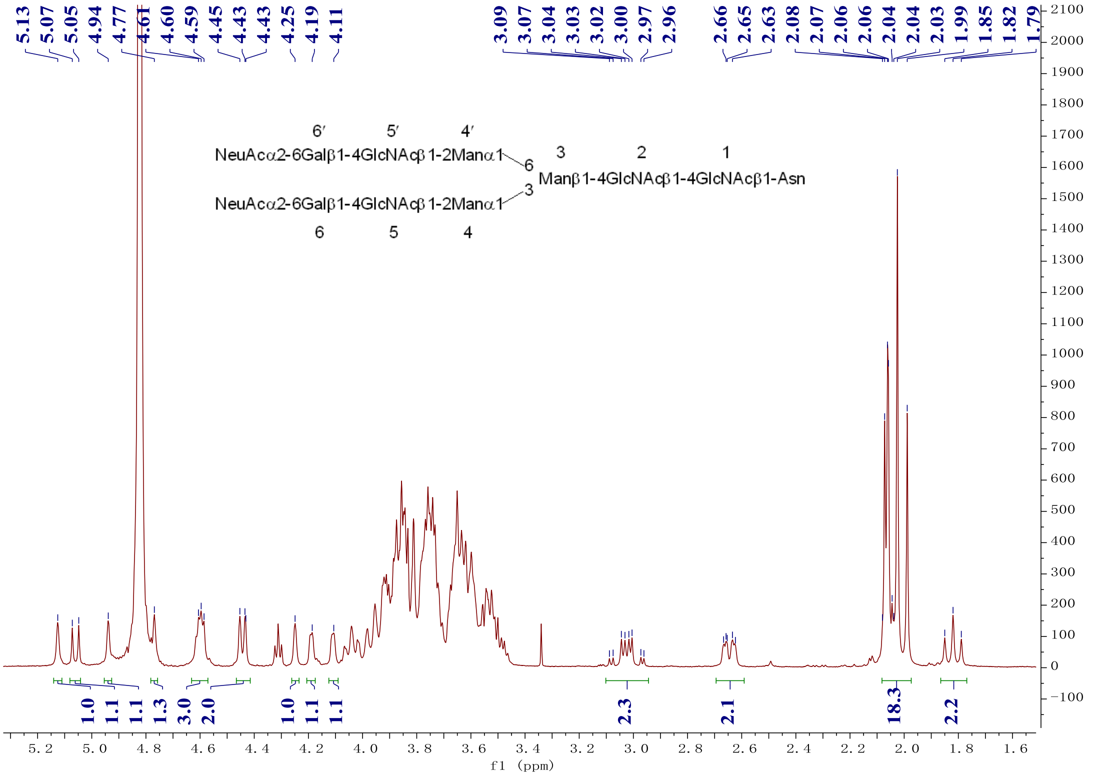

5. ^{13}C NMR (151 MHz, D_2O) spectrum of SCT-Asn

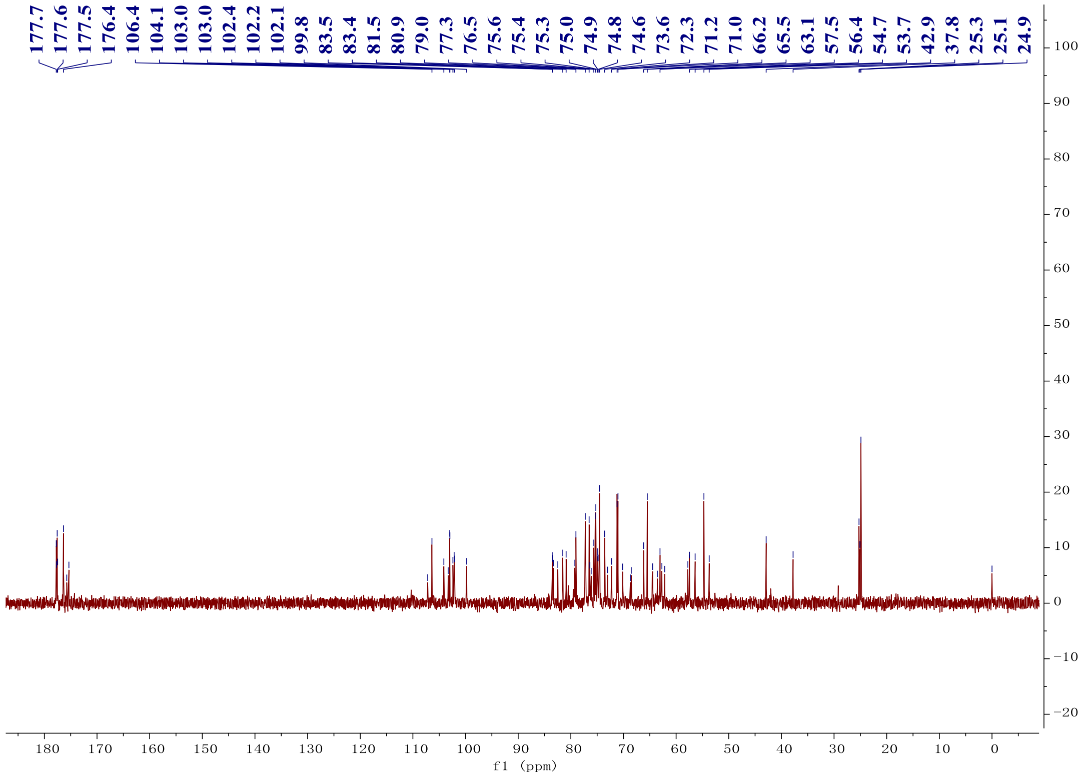

6.ESI-MS spectrum of SCT-Asn

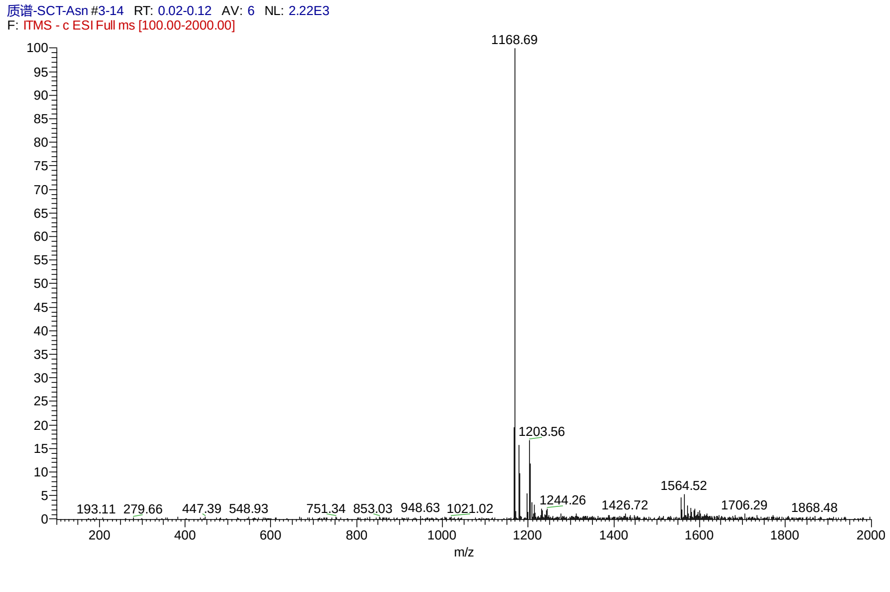

7. ^{1}H NMR (400 MHz, D_2O) spectrum of SCT-Asn-alkyne

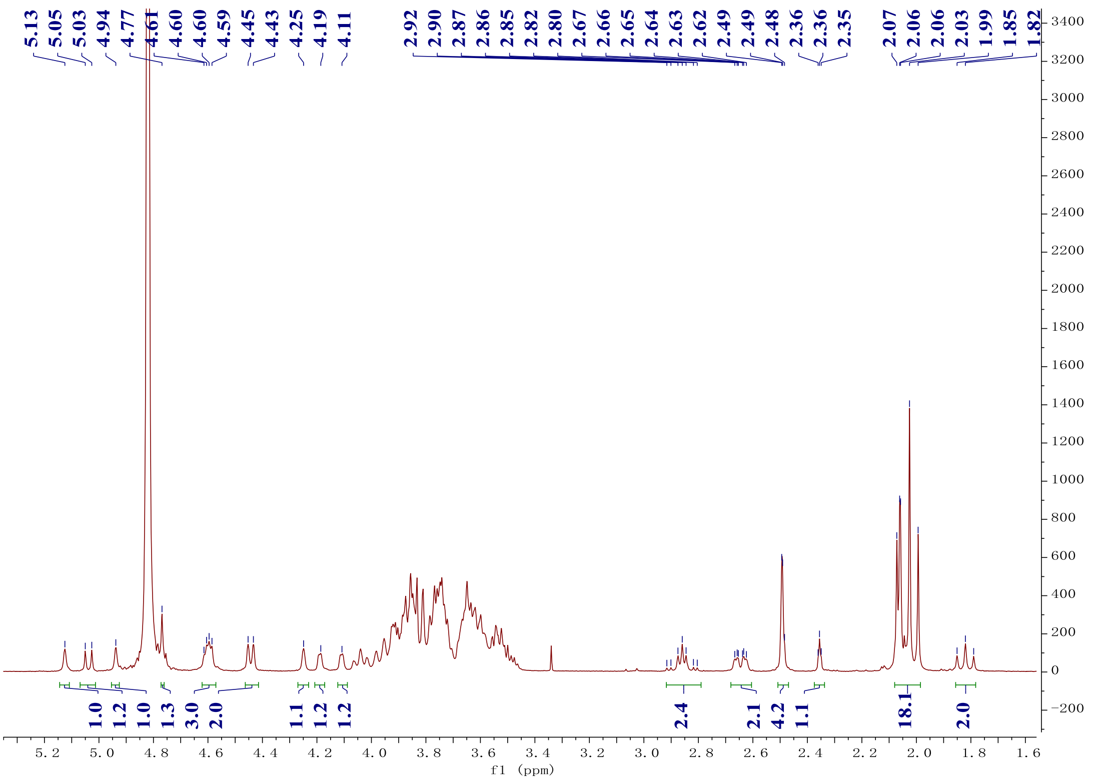

8. ^{13}C NMR (151 MHz, D_2O) spectrum of SCT-Asn-alkyne

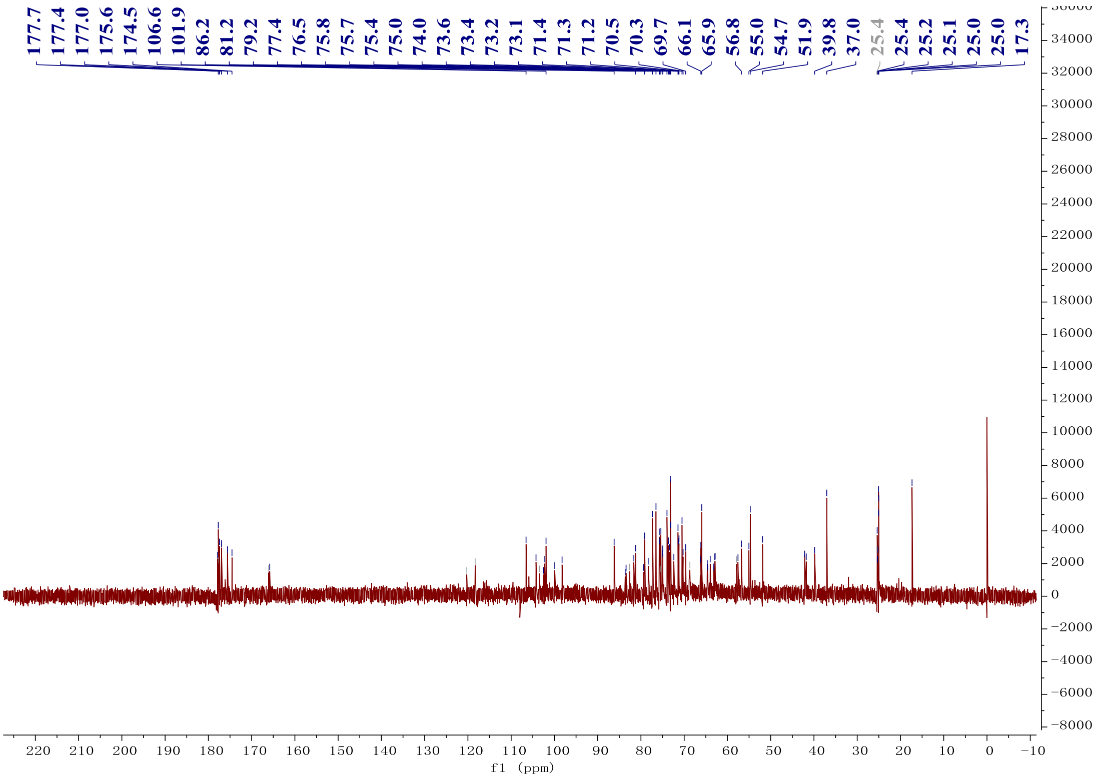

9. ESI-MS spectrum of SCT-Asn-alkyne

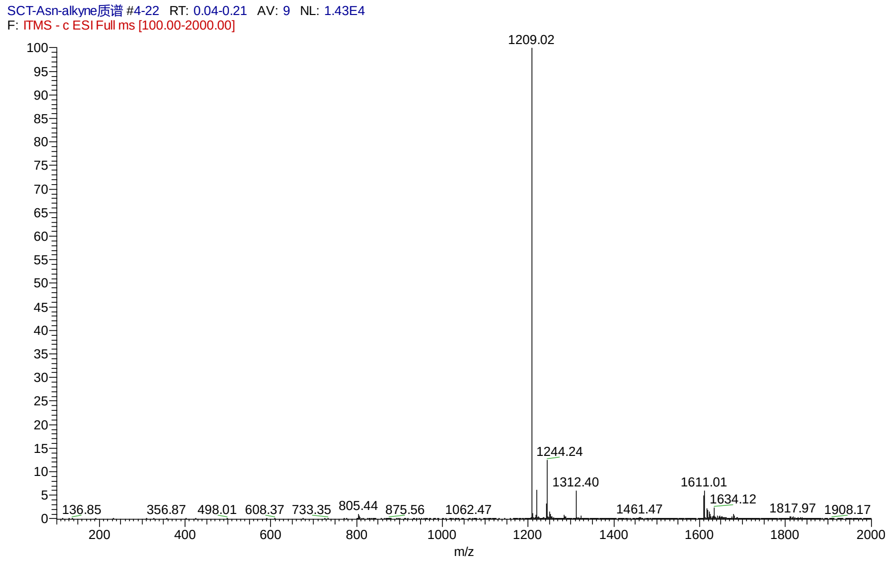

10. ^{1}H NMR (600 MHz, $CDCl_3$) spectrum of ursolic acid-N_3

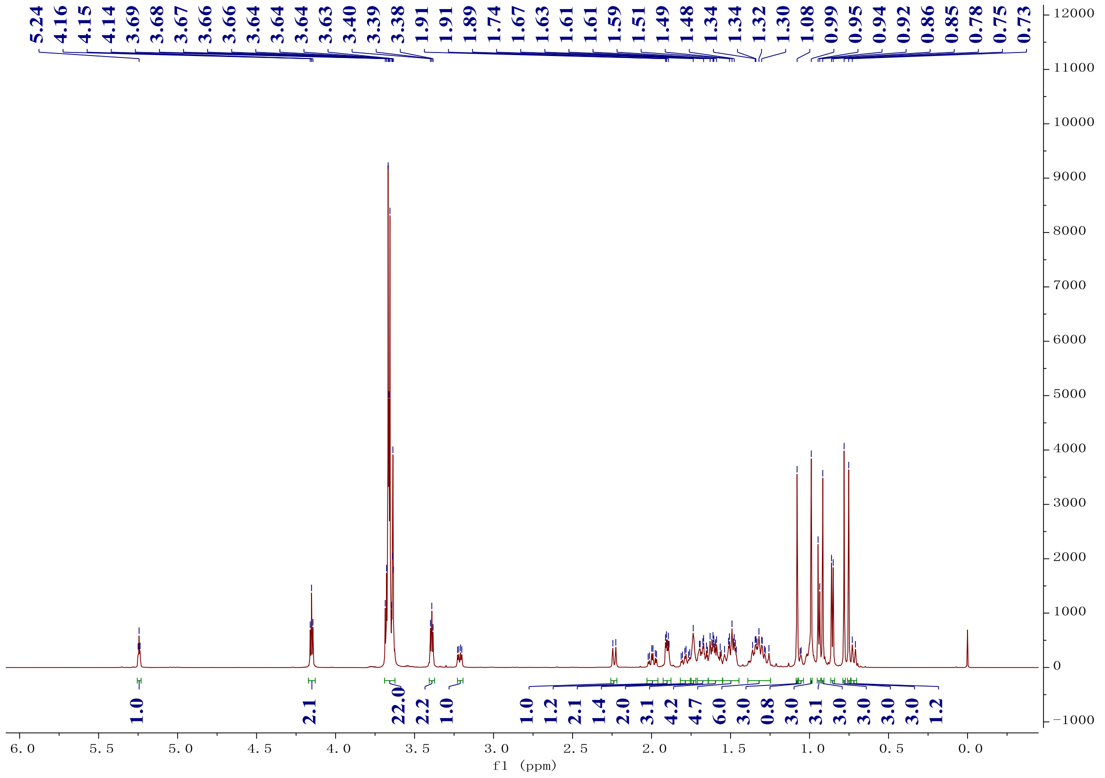

11. ^{13}C NMR (151 MHz, $CDCl_3$) spectrum of ursolic acid-N_3

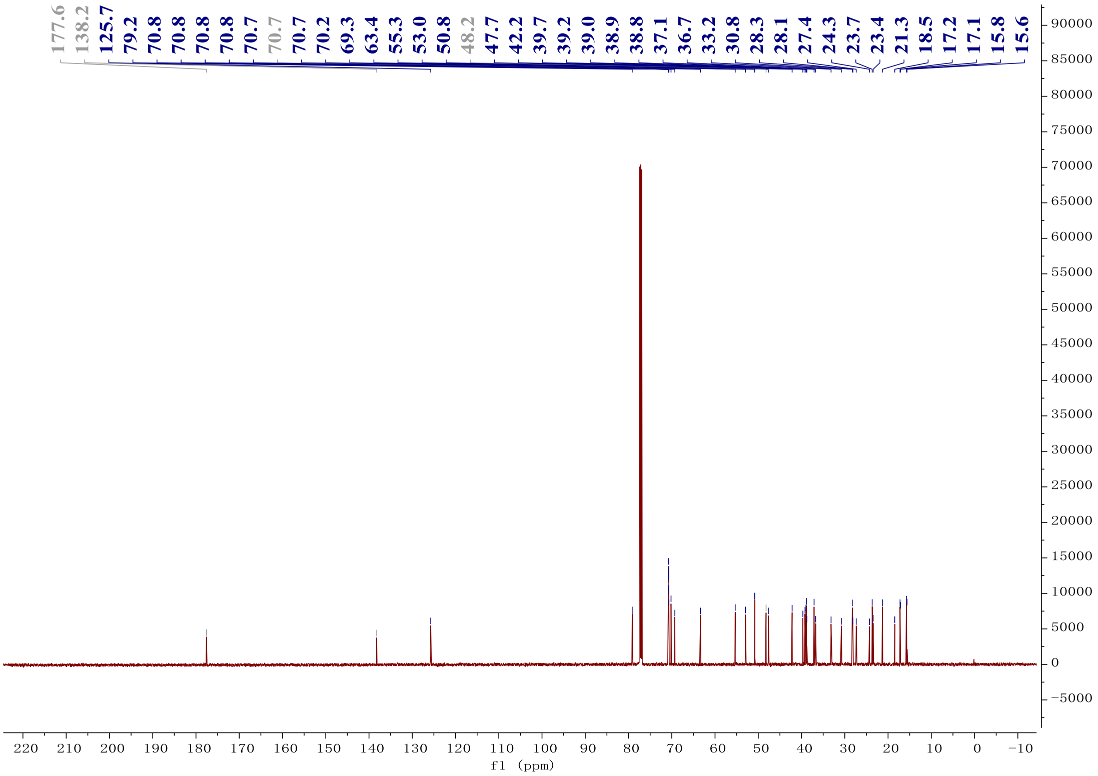

12. ESI-MS spectrum of ursolic acid-N_3

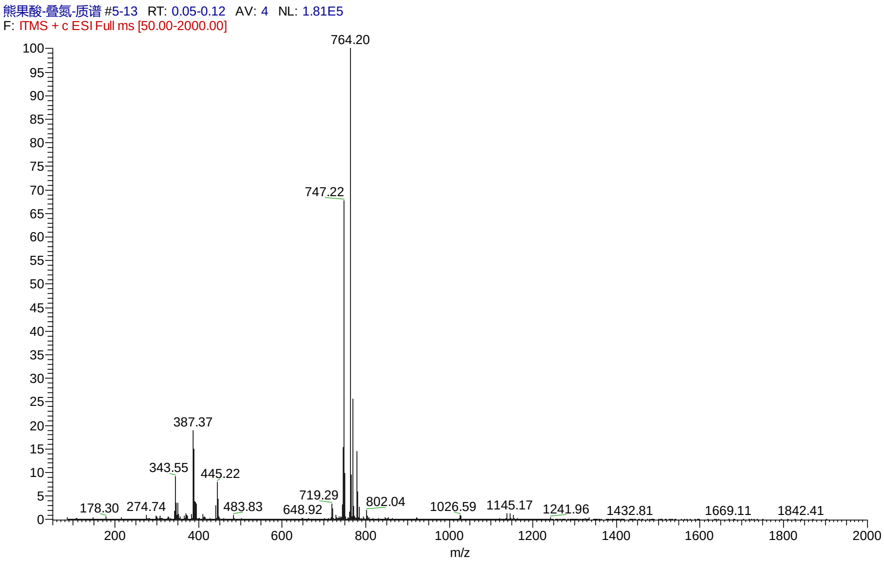

13. ^{1}H NMR (400 MHz, CD_3OD) spectrum of SCT-Asn-ursolic acid

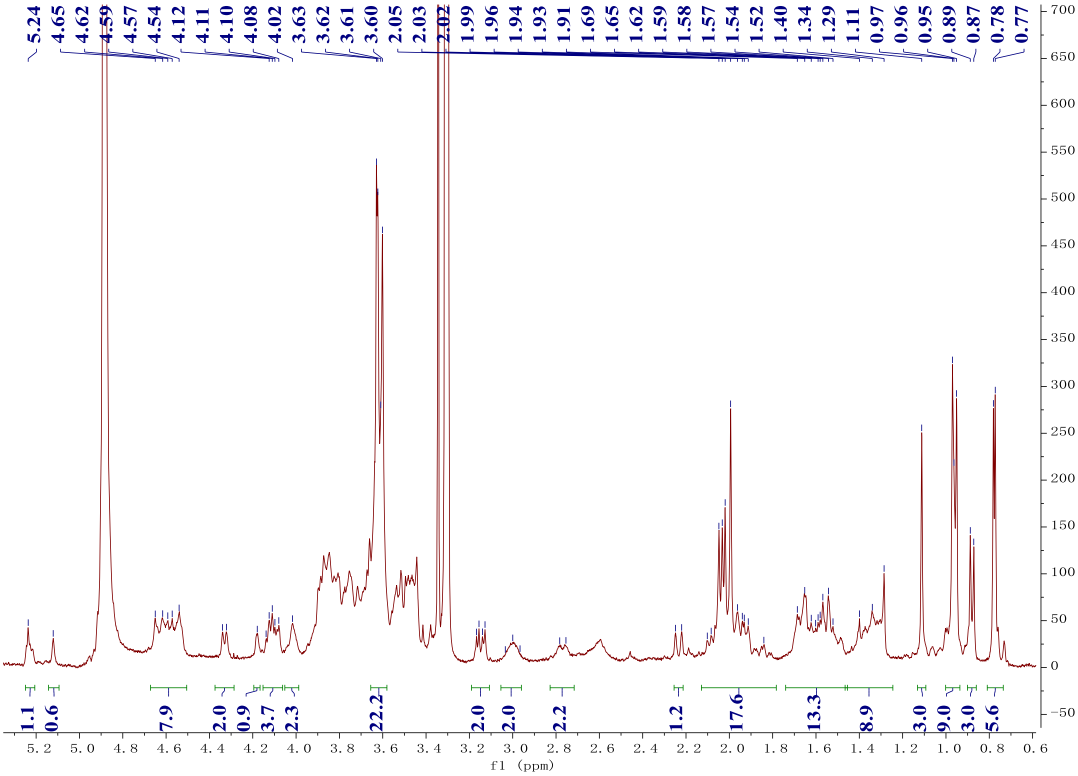

14. ESI-MS spectrum of SCT-Asn-ursolic acid

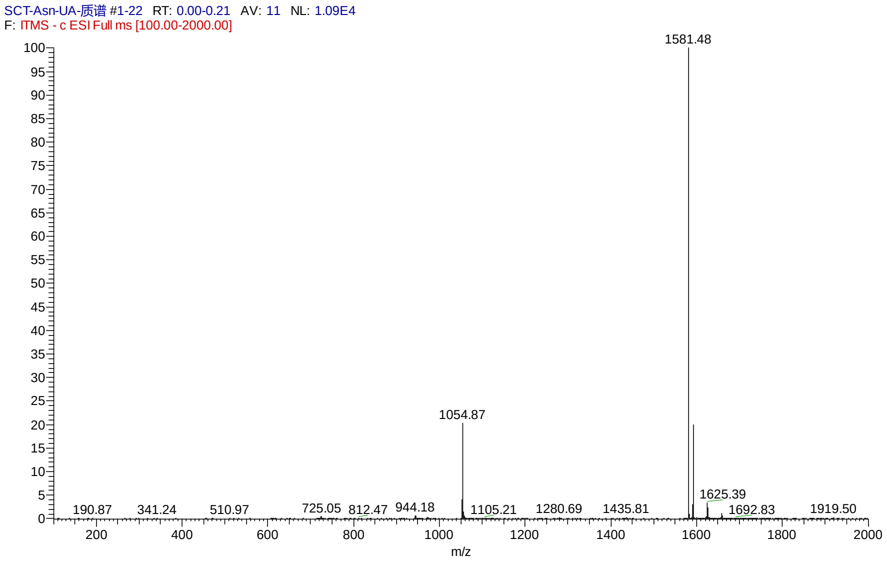

15. ^{1}H NMR (600 MHz, $CDCl_3$) spectrum of oleanolic acid-N_3

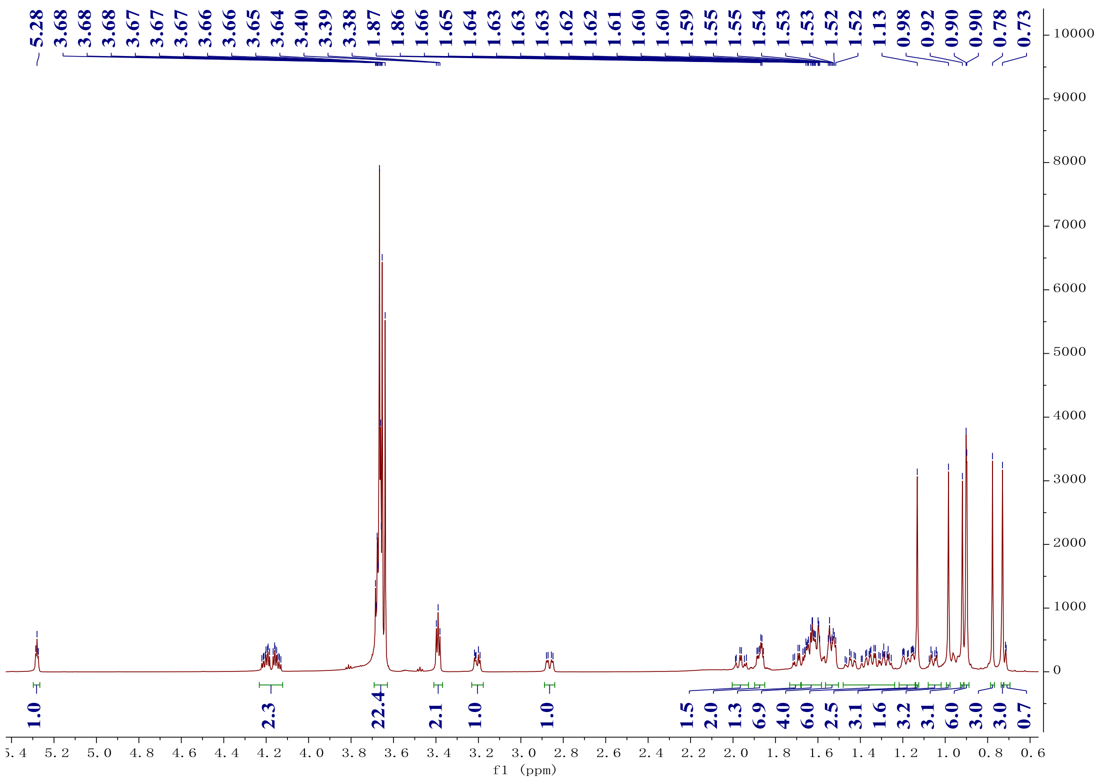

16. ^{13}C NMR (101 MHz, $CDCl_3$) spectrum of oleanolic acid-N_3

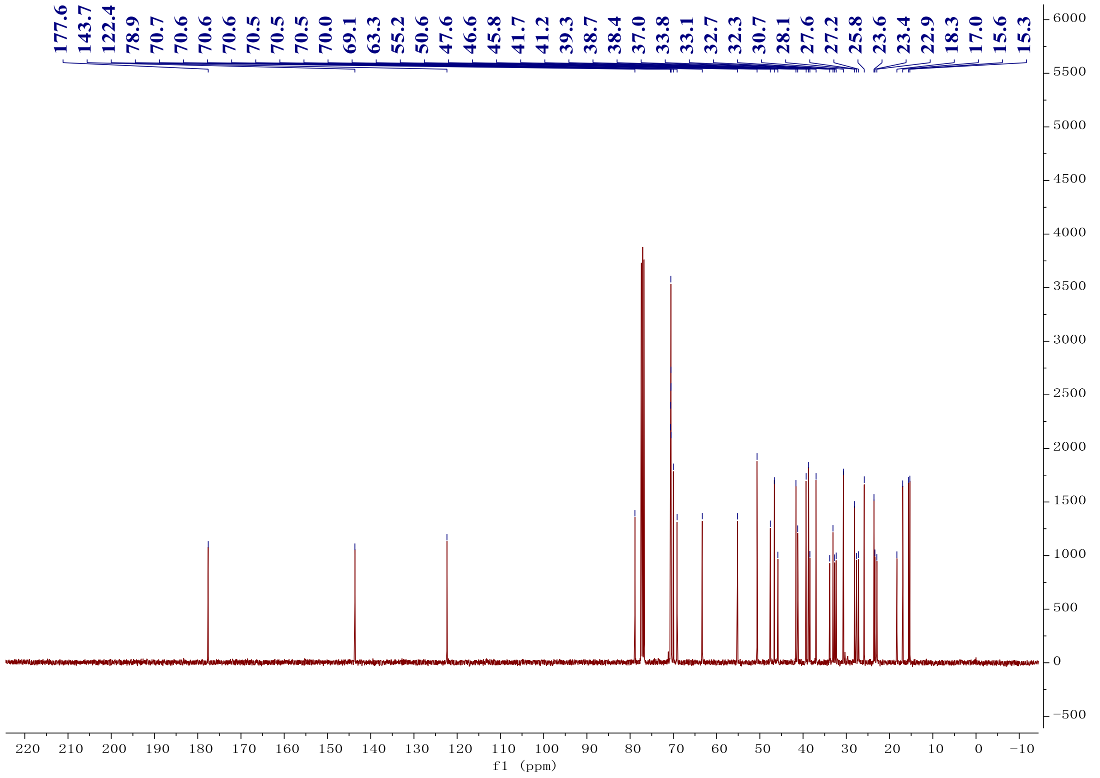

17. ESI-MS spectrum of oleanolic acid-N_3

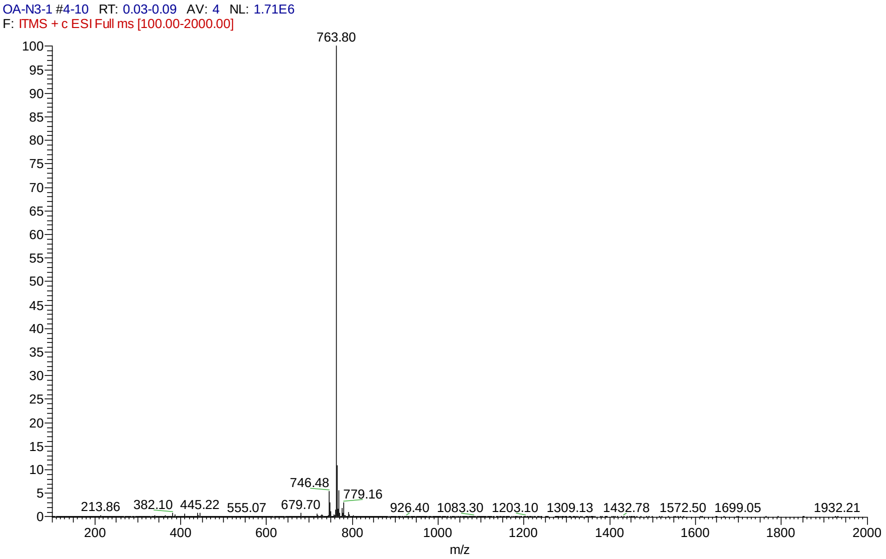

18. ^{1}H NMR (600 MHz, CD_3OD) spectrum of SCT-Asn-oleanolic acid

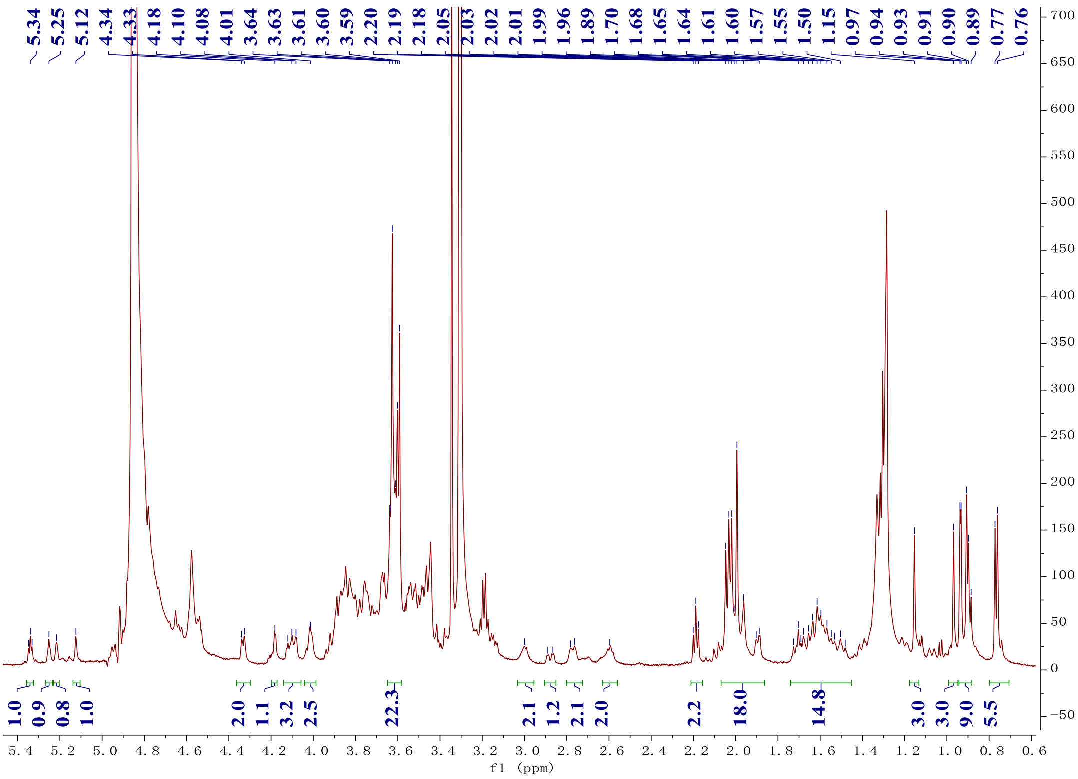

19. ESI-MS spectrum of SCT-Asn-oleanolic acid

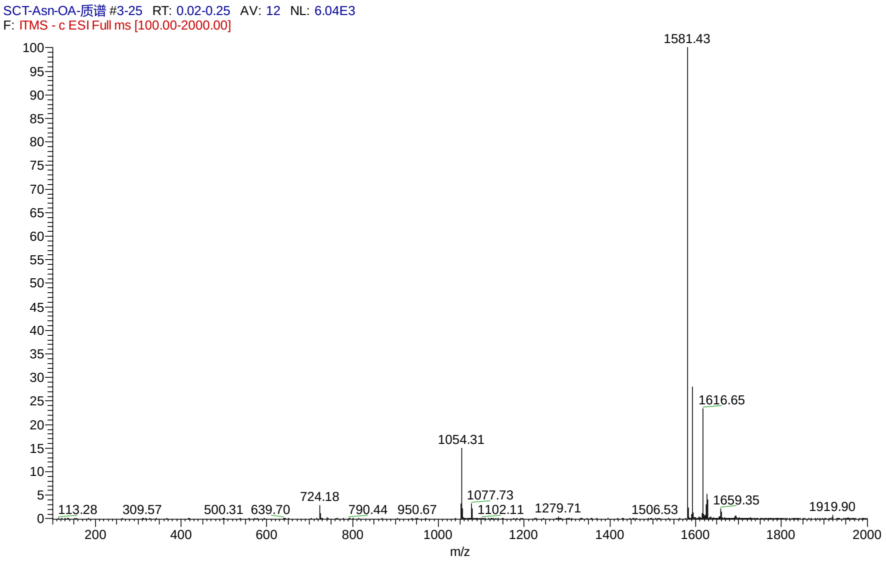

20. ^{1}H NMR (400 MHz, CD_3OD) spectrum of betulinic acid-N_3

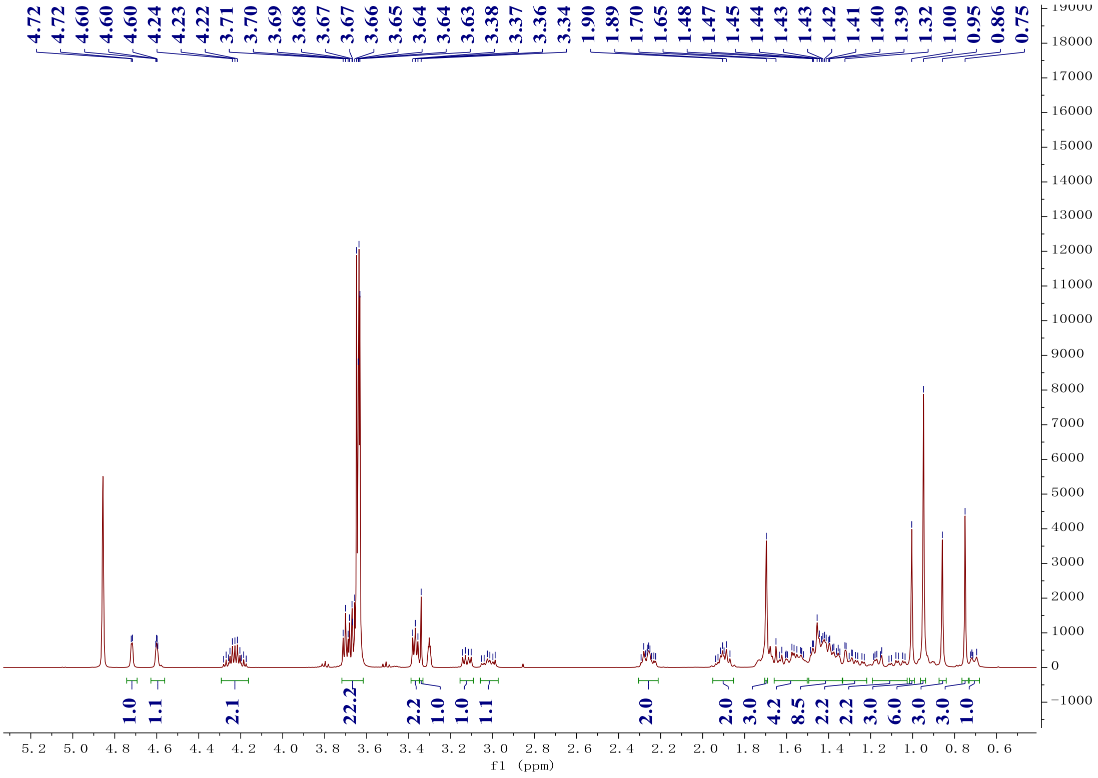

21. ^{13}C NMR (101 MHz, CD_3OD) spectrum of betulinic acid-N_3

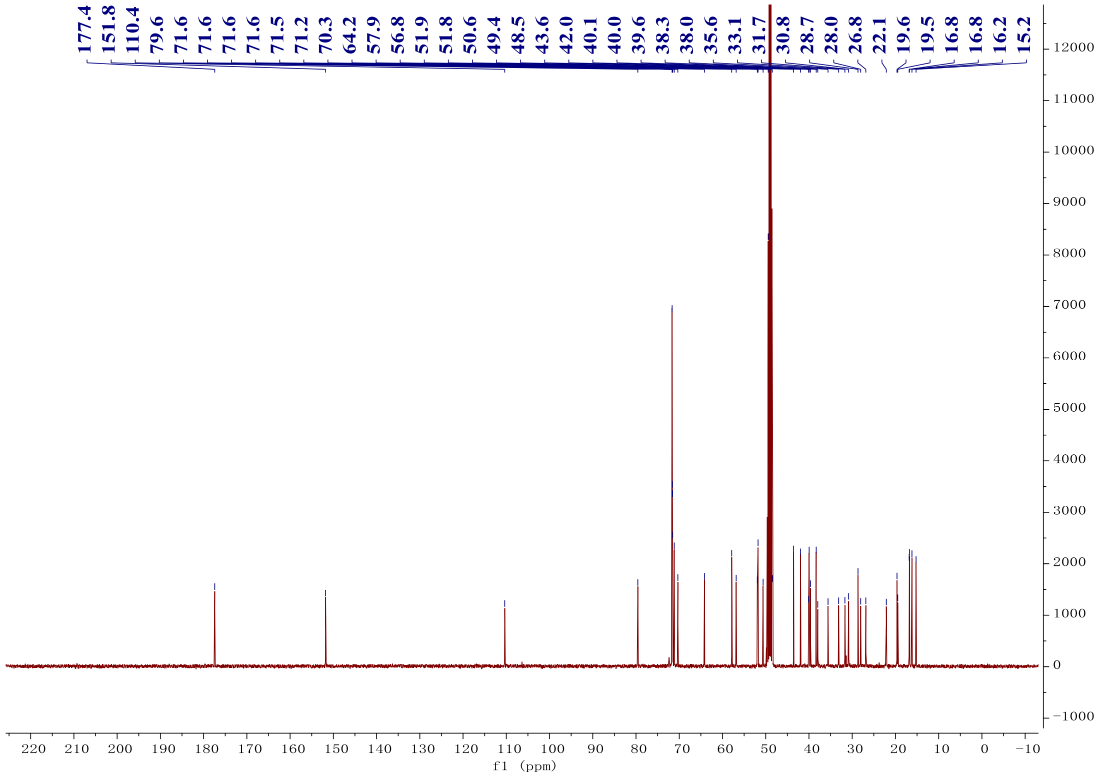

22. ESI-MS spectrum of betulinic acid-N_3

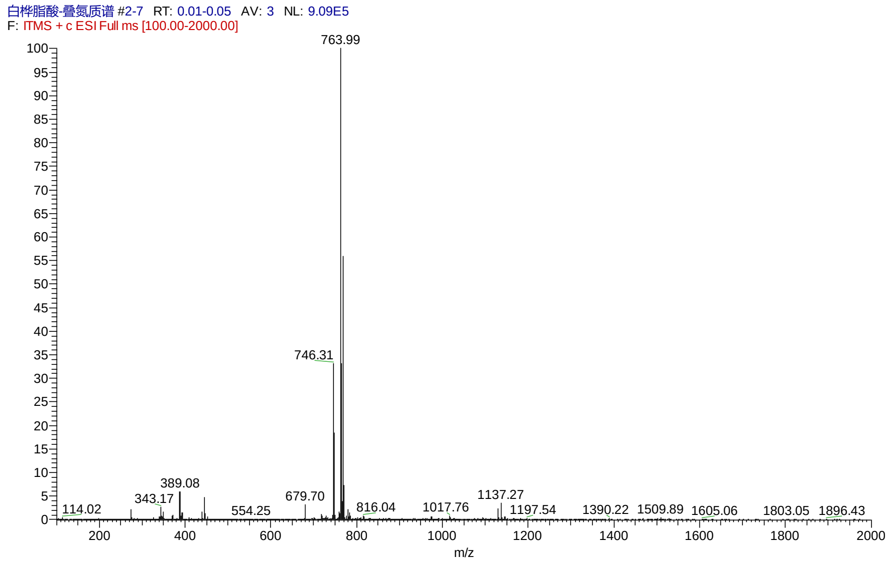

23. ^1H NMR (600 MHz, CD_3OD) spectrum of SCT-Asn-betulinic acid

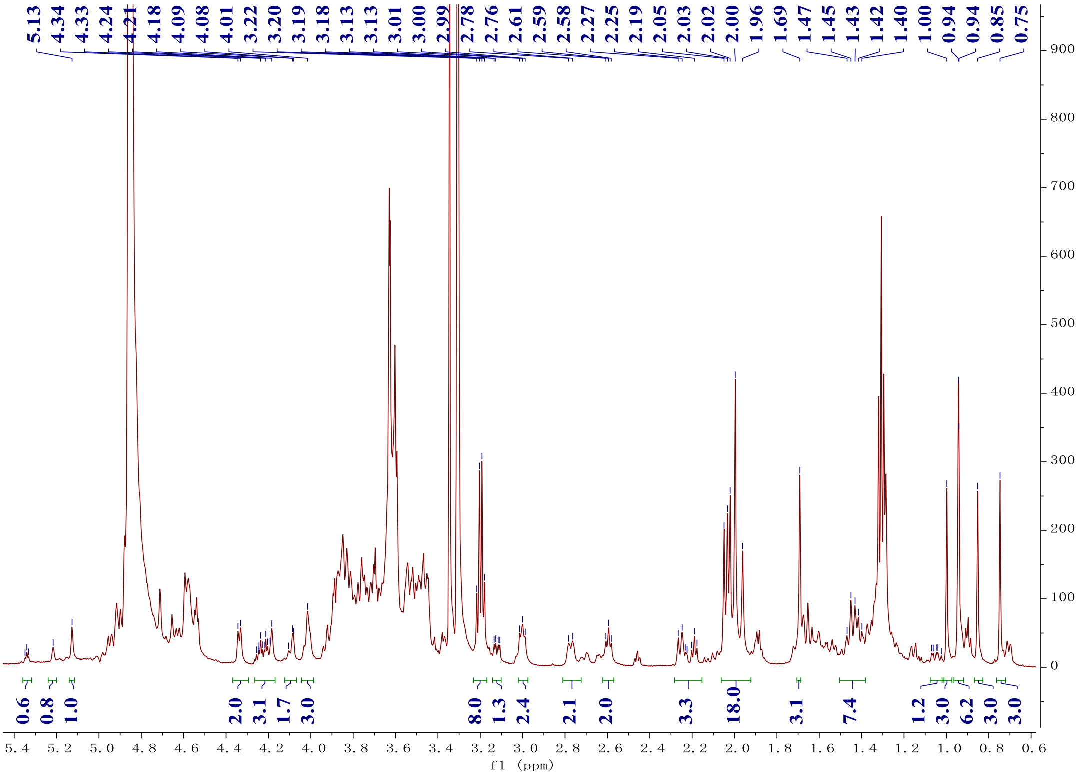

24. ESI-MS spectrum of SCT-Asn-betulinic acid

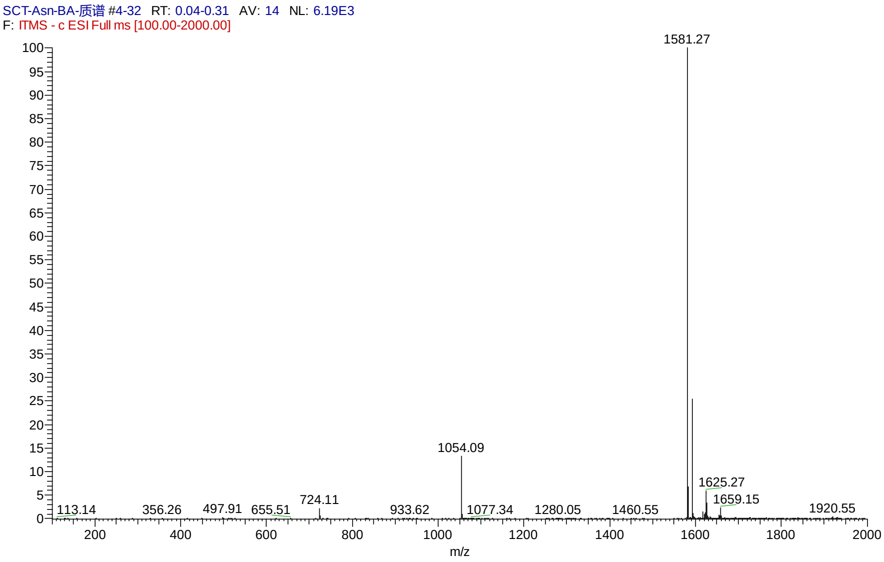

25. ^{1}H NMR (400 MHz, CD_3OD) spectrum of glycyrrhetinic acid-N_3

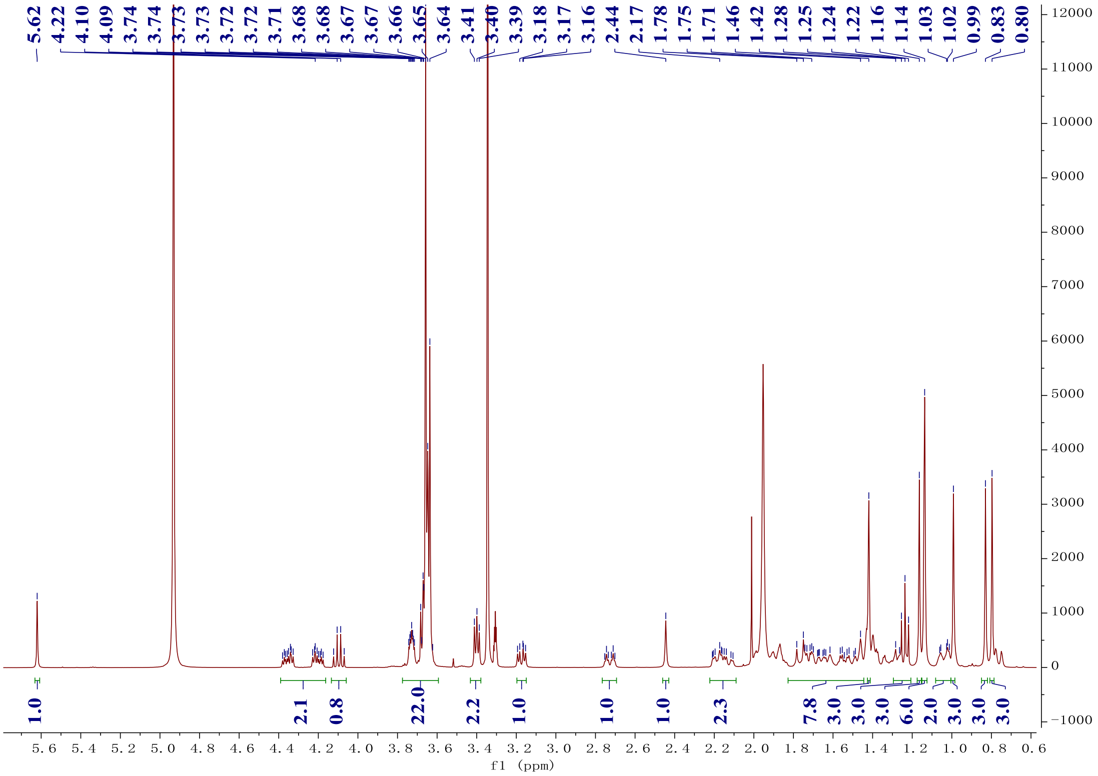

26. ^{13}C NMR (101 MHz, CD_3OD) spectrum of glycyrrhetinic acid-N_3

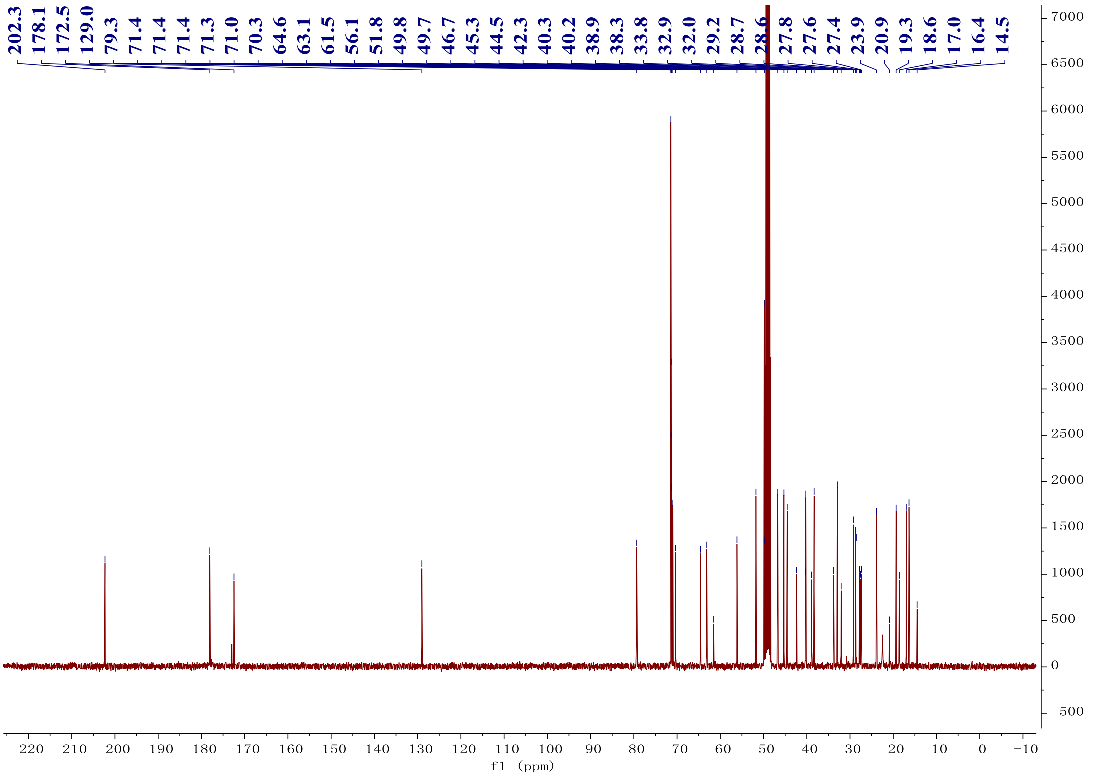

27. ESI-MS spectrum of glycyrrhetinic acid-N_3

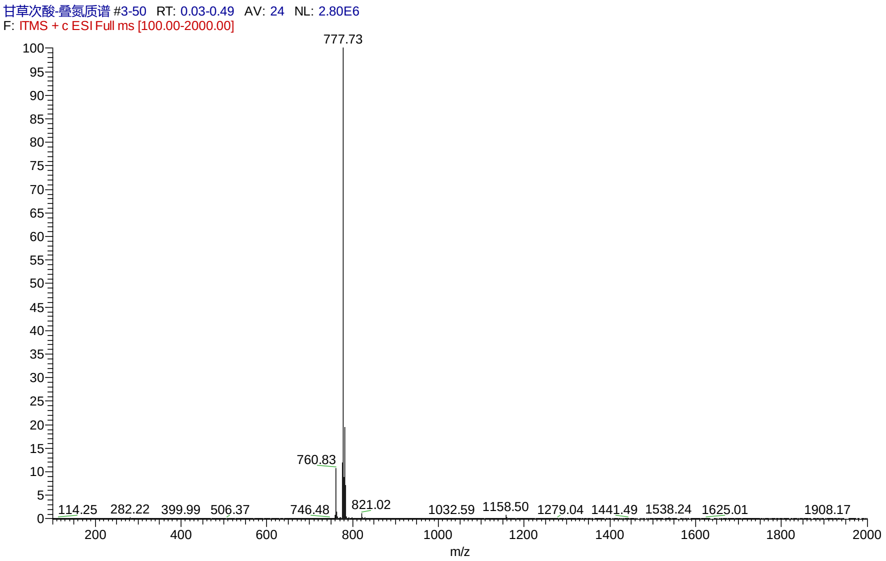

28. ^1H NMR (600 MHz, CD_3OD) spectrum of SCT-Asn-glycyrrhetinic acid

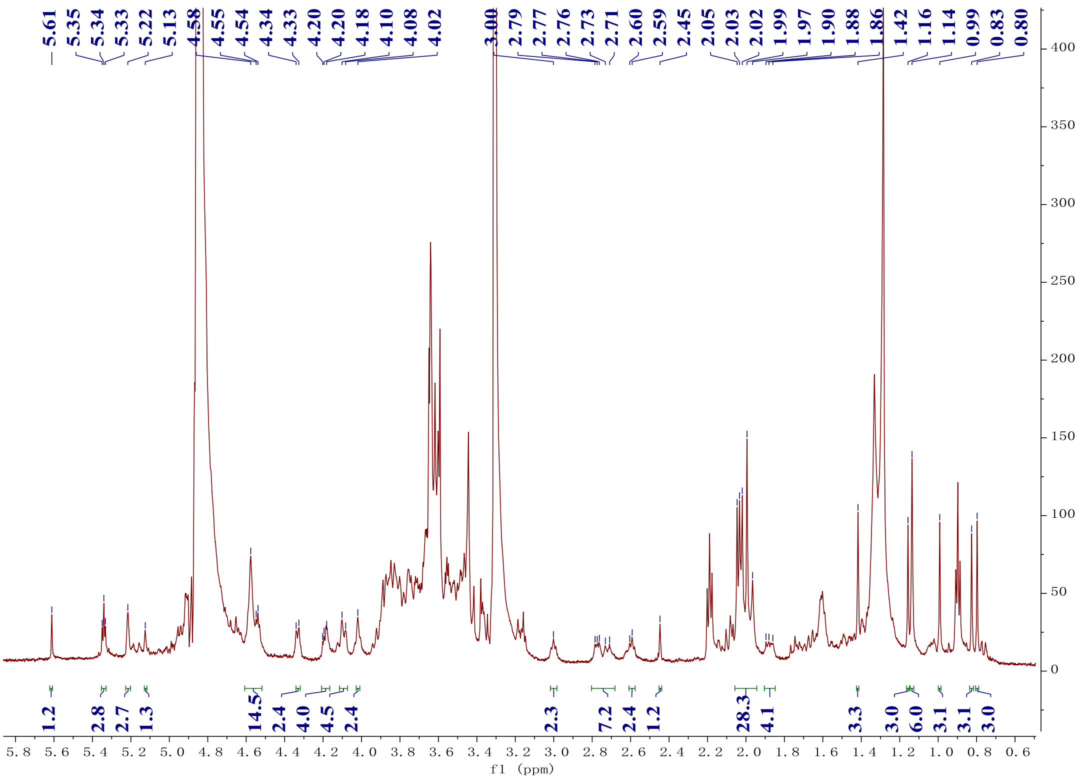

29. ESI-MS spectrum of SCT-Asn-glycyrrhetinic acid

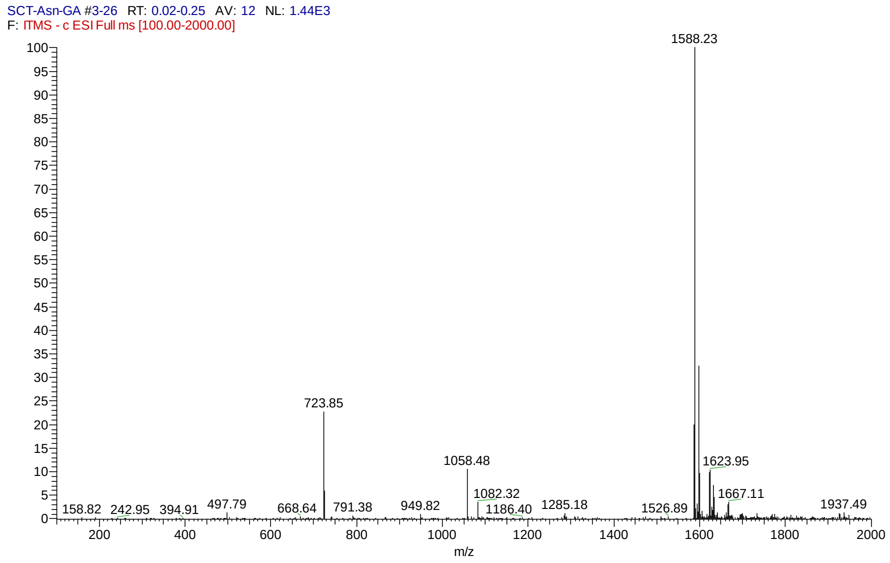

High resolution mass spectra of four conjugates.

1. HR-ESI-MS spectrum of SCT-Asn-UA

Qualitative Analysis Report

**Data Filename**	ESIH202100686.d	**Sample Name**	SCT-Asn-UA
**Sample ID**		**Position**	P1-B5
**Instrument Name**	Agilent G6520 Q-TOF	**Acq Method**	20160322_MS_ESIH_POS_1min.m
**Acquired Time**	1/28/2021 18:16:31	**IRM Calibration Status**	Success
**DA Method**	small molecular data analysis method.m	**Comment**	ESIH by ZZY

User Spectra

Fragmentor Voltage	Collision Energy	Ionization Mode
135	0	ESI

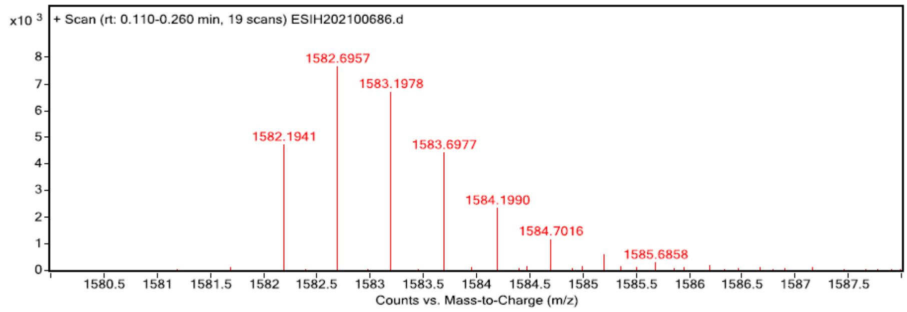

Formula Calculator Results

m/z	Calc m/z	Diff (mDa)	Diff (ppm)	Ion Formula	Ion
1582.1941	1582.1954	1.27	0.81	C135 H221 N11 O73	(M+2H)+2

2. HR-ESI-MS spectrum of SCT-Asn-OA

Qualitative Analysis Report

**Data Filename**	ESIH202100688.d	**Sample Name**	SCT-Asn-OA
**Sample ID**		**Position**	P1-B7
**Instrument Name**	Agilent G6520 Q-TOF	**Acq Method**	20160322_MS_ESIH_POS_1min.m
**Acquired Time**	1/28/2021 18:19:28	**IRM Calibration Status**	Success
**DA Method**	small molecular data analysis method.m	**Comment**	ESIH by ZZY

User Spectra

Fragmentor Voltage	Collision Energy	Ionization Mode
135	0	ESI

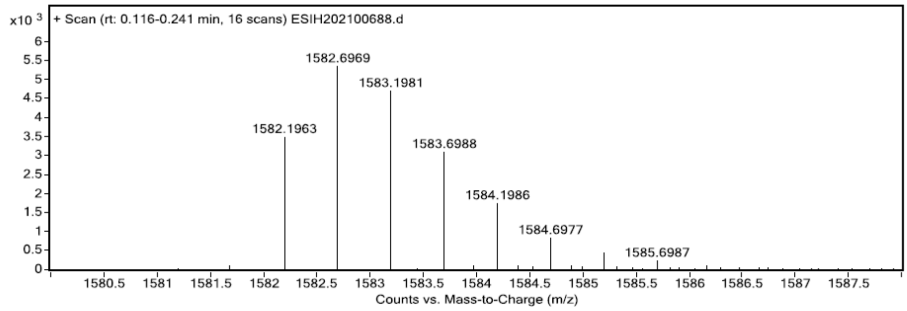

Formula Calculator Results

m/z	Calc m/z	Diff (mDa)	Diff (ppm)	Ion Formula	Ion
1582.1963	1582.1954	-0.91	-0.57	C135 H221 N11 O73	(M+2H)+2

3. HR-ESI-MS spectrum of SCT-Asn-BA

Qualitative Analysis Report

**Data Filename**	ESIH202100687.d	**Sample Name**	SCT-Asn-BA
**Sample ID**		**Position**	P1-B6
**Instrument Name**	Agilent G6520 Q-TOF	**Acq Method**	20160322_MS_ESIH_POS_1min.m
**Acquired Time**	1/28/2021 18:18:00	**IRM Calibration Status**	Success
**DA Method**	small molecular data analysis method.m	**Comment**	ESIH by ZZY

User Spectra

Fragmentor Voltage	Collision Energy	Ionization Mode
135	0	ESI

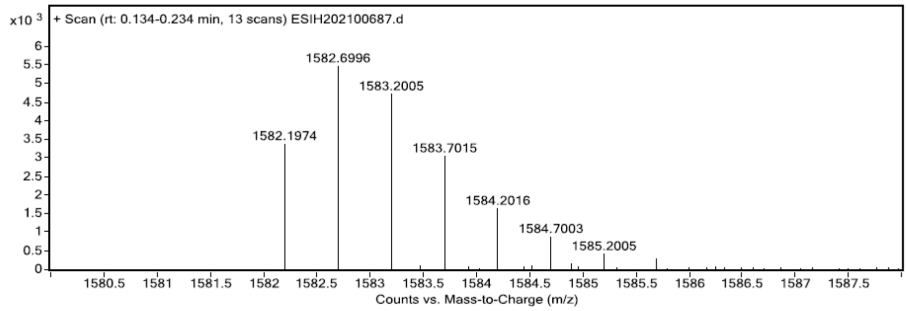

Formula Calculator Results

m/z	Calc m/z	Diff (mDa)	Diff (ppm)	Ion Formula	Ion
1582.1974	1582.1954	-1.97	-1.24	C135 H221 N11 O73	(M+2H)+2

4. HR-ESI-MS spectrum of SCT-Asn-GA

Qualitative Analysis Report

**Data Filename**	ESIH202100689.d	**Sample Name**	SCT-Asn-GA
**Sample ID**		**Position**	P1-B8
**Instrument Name**	Agilent G6520 Q-TOF	**Acq Method**	20160322_MS_ESIH_POS_1min.m
**Acquired Time**	1/28/2021 18:20:57	**IRM Calibration Status**	Success
**DA Method**	small molecular data analysis method.m	**Comment**	ESIH by ZZY

User Spectra

Fragmentor Voltage	Collision Energy	Ionization Mode
135	0	ESI

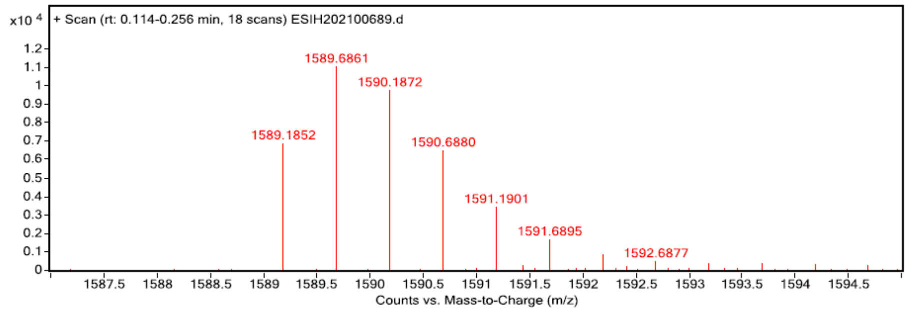

Formula Calculator Results

m/z	Calc m/z	Diff (mDa)	Diff (ppm)	Ion Formula	Ion
1589.1852	1589.185	-0.15	-0.1	C135 H219 N11 O74	(M+2H)+2
